# Fibroblast activation protein (FAP)-mediated promotion of metastasis via the FN1-TGFβ axis and immune suppression in aggressive thyroid cancer

**DOI:** 10.1186/s12967-025-07307-3

**Published:** 2025-11-13

**Authors:** Mario Udinotti, Udo Siebolts, Marcus Bauer, Christoforos Vaxevanis, Antonios Asiminas, Kerstin Lorenz, Christine Dierks, Claudia Wickenhauser, Barbara Seliger

**Affiliations:** 1 Institute of Pathology, Martin Luther University Halle-Wittenberg, Halle, Germany; 2 Institute of Pathology, University Hospital, Cologne, Germany; 3 Department of Visceral-, Vascular, and Endocrine Surgery, Martin Luther University Halle-Wittenberg, Halle, Germany; 4 Department of Hematology/Oncology and Stem Cell Transplantation, KIM IV, Martin-Luther University Halle-Wittenberg, Halle, Germany; 5 Department of Hematology and Oncology, Freiburg University Medical Center, Albert-Ludwigs-University of Freiburg, Breisgau, Germany; 6 Center for Translational Neuromedicine, University of Copenhagen, Copenhagen, Denmark; 7 Institute of Translational Immunology, Center for Translational Medicine, Faculty of Health Sciences Brandenburg, Brandenburg Medical School Theodor Fontane, Brandenburg, Germany; 8 Fraunhofer Institute for Cell Therapy and Immunology (IZI), Leipzig, Germany

**Keywords:** Thyroid cancer, Tumor microenvironment, Extracellular matrix components, Immune escape, Invasion, Cancer associated fibroblasts, Fibroblast activating protein

## Abstract

**Background:**

Treatment options of aggressive thyroid carcinoma (TC) is limited due to its aggressiveness. Therefore, increased knowledge of the role of tumor intrinsic immune escape mechanisms and the microenvironment contributing to metastases formation is essential to improve the patients’ outcome. These include extracellular matrix (ECM) components, cancer-associated fibroblasts (CAFs) and the expression of the fibroblast activation protein (FAP) known to be involved in immune evasion, disease progression and a worse prognosis of thyroid carcinoma (TC) patients.

**Methods:**

The Cancer Genome Atlas (TCGA) and Human Protein Atlas datasets of TC specimens as well as tissue microarrays (TMA) consisting of 187 TC samples were analyzed for marker expression and immune cell infiltration. TC cell lines treated with recombinant FAP, a FAP inhibitor, transfected with siRNA-FAP plasmids and/or co-cultured with CAFs were subjected to qPCR, Western blot analyses and flow cytometry, cell supernatants to ELISA for cytokine secretion, the TMAs to conventional immunohistochemistry (IHC).

**Results:**

Immunohistochemical staining and bioinformatics analyses of publicly available mRNA and protein expression profiles revealed a significant positive correlation between high FAP and fibronectin 1 (FN1) expression, metastatic parameters and CD8^+^ T cell infiltration in TC lesions compared to adjacent normal tissues. FAP directly cleaved FN1 associated with lower adhesion, a high invasive TC phenotype and inhibition of transforming growth factor beta (TGF-β). This is accompanied by reduced programmed death ligand 1 (PD-L1) and increased human leukocyte antigen (HLA)-ABC surface levels. Co-culture of TC cells with CAFs demonstrated an influence of FAP on the immune suppressive CAF subtypes.

**Conclusions:**

FAP-mediated alterations of ECM integrity, TC cells and CAFs underscore its potential as a powerful marker for tumor immunogenicity, immune infiltration and metastasis rendering it a promising therapeutic target for the treatment of aggressive ATC.

**Supplementary information:**

The online version contains supplementary material available at 10.1186/s12967-025-07307-3

## Background

Malignant neoplasia of the thyroid gland encompasses differentiated, poorly differentiated and undifferentiated (anaplastic) thyroid carcinoma (TC) with distinct histopathological and clinical features that are associated with variable treatment options and survival probabilities [1, 2]. Anaplastic thyroid carcinoma (ATC) is a highly aggressive form of TC with a rapid progression, resistance to treatment and a very poor patients’ prognosis. Despite advances in targeted therapies for other TC, effective treatment of ATC remains a challenge suggesting an urgent need for novel therapeutic strategies [3–5]. This might be mediated by the modulation of the intricate interplay between tumor cells and the tumor microenvironment (TME), in particular the surrounding stroma and the immune cell composition [6]. During the last decade, the fibroblast activation protein (FAP), a cell surface glycoprotein, has emerged as a pivotal player in orchestrating key aspects of tumorigenesis, immune modulation and metastatic progression [7]. Based on its diverse functions and frequent overexpression in tumors, FAP has rekindled interest as a prognostic biomarker and therapeutic target [6]. In both differentiated and undifferentiated TC, FAP is involved in disease progression due to its involvement in ECM remodeling and its association with fibronectin (FN1), a major extracellular matrix (ECM) component involved in tumor cell adhesion, migration [8–10] and the acquisition of mesenchymal characteristics [11].

Concerning ATC, FAP has been suggested to play a significant role in immune escape strategies [12–14], such as downregulation of human leukocyte antigen class I (HLA-I) antigens [15] and an upregulation of immune checkpoint (ICP) molecules, e.g., the programmed death ligand 1 (PD-L1, CD274), known for its role in dampening anti-tumor immune responses [16, 17]. In addition, it modulates the complex network of metastatic signaling pathways, which is in particular a characteristic of aggressive ATC [18].

Since a better understanding of FAP in progression and immune surveillance in ATC is required to improve therapeutic options for this disease, this study aims to decipher the role of FAP in regulating epithelial mesenchymal transition (EMT), the expression of metastasis-associated molecules and immune modulatory molecules across various TC subtypes with a specific focus on ATC. This will be complemented by in silico analysis of The Cancer Genome Atlas (TCGA) and Human Protein Atlas datasets, immunohistochemical staining of TC specimens as well as co-culture experiments of TC cells with fibroblasts and their treatment with recombinant FAP or FAP inhibitor (Figure S1). A FAP-mediated promotion of metastasis via the FN1-TGF-β axis accompanied by a reduced immunogenicity was identified in ATC and provides the basis for the development of targeted therapies to increase treatment efficacy of this disease.

## Methods

### Cell culture and treatment with drugs

TC cell lines of distinct subtypes were kindly provided by Prof. Dr. Cuong Hoàng-Vu (Laboratory Surgical Research, University Hospital Halle, Halle, Germany, and Prof. Dr. Stefan Huettelmaier, Medical Faculty of the Martin Luther University Halle-Wittenberg, Institute for Molecular Medicine, Section Molecular Cell Biology, Halle, Germany). These include the ATC cell lines 8305C, 8505C, BHT101, C643, Hth74, the follicular thyroid carcinoma (FTC) cell line FTC-133, the papillary thyroid carcinoma (PTC) cell line BCPAP and the normal thyroid cell line Nhty-ori-3–1. The BJ fibroblast cell line was purchased from American Type Culture Collection (ATCC) (Manassas, USA). All TC cell lines were cultured in Roswell Park Memorial Institute 1640 (RPMI), the BJ fibroblast cell line in Eagle’s minimum essential medium (EMEM) supplemented with 10% fetal bovine serum (FBS) (Gibco, Waltham, USA), 1% L-glutamine (Sigma-Aldrich, Burlington, USA) and 1% penicillin-streptomycin (Sigma-Aldrich). Adherent cells were detached using trypsin-EDTA (0.25%) (Gibco, Waltham, USA). Cell cultures were maintained in T75 flasks in a humidified incubator at 37 °C with 5% CO2.

For spheroid formation, 1x10^6^ cells/well were seeded into the ultra-low attachment plates (CORNING, New York, USA) supplemented with 2 ml of complete medium RPMI and spheroid formation was analyzed over a three-day culture period.

For FAP inhibition, cells were treated with 10 μM of the FAP inhibitor (FAPi) BR103354 (AOBIUS, Massachusetts, USA). Treatment of cells with recombinant FAP (rFAP; 300 ng/ml) or 400 ng/ml recombinant TGF-β (Biolegend), respectively, was performed for the time points indicated.

### Transfection of TC cells

For transfection, 70–80% confluent TC cells were subjected to lipofection using lipofectamine 2000 for the FAP-tGFP and pmR-mCherry plasmid and lipofectamine RNAiMAX (Invitrogen, Darmstadt, Germany) for siFAP (Thermo Scientific, Bremen, Germany) and siRNA negative control (Thermo Scientific, Bremen, Germany), respectively. Both lipofectamines were mixed with the respective plasmid DNA/siRNA according to the manufacturer’s instructions and then added to the cells for 48 hours, before the cells were harvested and subjected to further analysis.

### Quantitative RT-PCR

Total cellular mRNA was isolated using RNA isolation kit (Machery-Nagel) according to the manufacturers’ protocol. The cDNA using the ReverseAid First strand cDNA synthesis kit (Thermo Fisher) protocol and subjected to PCR using the BioRAD CFX qPCR cycler and a SYBRGreen PCR Master Mix (Vazyme). The primers used in this study were provided in Supplementary files (Table S1).

### Immunoblotting

Total cellular protein was extracted and protein concentrations were determined using the Piece BCA protein kit (Thermo Scientific). 10–30 µg protein/well were subjected to gel on electrophoresis and transferred onto iblot polyvinylidene difluoride (PVDF) or nitrocellulose (NC) membranes (Invitrogen) using an iBlot2 transfer system (Invitrogen). Membranes were blocked with 5% bovine serum albumin (BSA) for 1 hour at room temperature. The blots were then stained with primary antibodies (Ab) overnight at 4 °C followed by staining with horseradish peroxidase (HRP)-conjugated secondary antibodies (Cell Signaling Technology) for 1 hour at room temperature. Protein bands were visualized using an enhanced chemiluminescence (ECL) detection system (Cell signaling). Proteome profiler blots (RnD) were used according to the manufacturers’ protocol. Western blots and Proteome profilers were imaged and quantified using the iBright750 Imaging System (Invitrogen). Information of Abs used for Western blot analysis were provided in Supplementary files (Table S1).

### 3D live cell imaging

For live cell imaging pictures and quantification, the Pathway 855 bioimaging system (Becton Dickinson) was used. The well plates with spheroids in different conditions were inserted into the microscope’s incubator chamber. After ROI identification, the camera within the software interface is activated to capture images. The software facilitates the selection of wells within a different type of well plates and streamlines imaging processes with a macro-scheduling feature. Laser settings and channels are adjusted for fluorescence imaging, with parameters optimized for specific fluorophores. To capture 3D structures, Z-stack imaging sequences are utilized, allowing approximately 70 captures of each channel with 1 μm interval. Analysis via ImageJ for background subtraction and thresholding techniques are applied to distinguish signal from noise. Time-lapse spheroid imaging was conducted in low attachment plates formed between 48 and 120 hours, with and without treatment and was monitored using the transmitted channel on a consistent 0.09 ms exposure. For single/co-culture experiments involving TC cells and BJ fibroblasts, the green fluorescent protein (GFP) channel for carboxyfluorescein succinimidyl ester (CFSE)-labeled fibroblasts and Alexa594 channel for the α-FN1 antibody (Ab) conjugated with zenonAlexa594 were utilized. Additionally, spheroids transferred to normal attachment plates allowed to migrate for 24 hours. Control and rFAP (300 ng/ml) treatments were imaged using the Alexa594 channel for α-CD29 PE, Alexa488 channel for α-HLA-I FITC, Alexa647 channel for α-FN1 conjugated with zenonAlexa647, HOECHST channel for Hoechst 33,342 and transmitted light. Zenon Alexa Abs (Invitrogen) were used to conjugate unlabeled Ab following the protocol of the manufacturer. Consistent exposure times were maintained across all channels with 5 μl/Ab/per stain. Threshold definitions for quantification were globally set in ImageJ software. All antibodies for live cell imaging are described in supplementary files (Table S1).

### Co-culture of TC cells with fibroblasts

For 2D co-culture experiments, 2x10^4^ TC cells and BJ fibroblasts/well were seeded at a 1:1 ratio in a 24-well plate. For 3D co-culture experiments, a 1:2 ratio/well of TC cells to naïve fibroblasts were used. Prior to seeding BJ fibroblasts were labeled with CFSE. Co-cultures were incubated under standard conditions for distinct time points and subjected to respective analysis. Information about all materials for co-culture experiments purchased were provided in Supplementary files (Table S1).

### Migration and invasion assays

Migration assays were performed using μ-dish with cross inserts (Ibidi, Gräfelfing, Germany) seeded with the appropriate cell number according to the manufacturer’s instructions until confluence was achieved. The seeded plates were then transferred to a BioStation IM-Q device (Nikon, New York, USA) to capture images at specified intervals over a 24 hours period under different conditions: vehicle (0.1% DMSO) vs. FAP inhibition (10 μM) and control vs rFAP (300 ng/ml). Images of biological replicates at the start and end were analyzed and quantified using ImageJ software.

Invasion assays were conducted using VitroGel (TheWell bioscience, New Jersey, USA) in the presence and absence of 100 ng/ml rFN1. A thin layer (100 μl) of VitroGel 1:1 with medium lacking fetal calf serum (FCS) was solidified onto the surface of a trans-well insert (8 μm pore size). Medium containing vehicle, FAP inhibitor or rFAP was added both below (10% FCS) and on top (0% FCS) of the trans-well. 24 hours later, the migrating cells were stained with 0.5% crystal violet, photographed with CytoSmart2 device (Lonza, Basel, Switzerland), re-dissolved into a new well plate using 1% SDS solution and absorbance at 570 nm was measured using a TECAN spectrophotometer (Männedorf, Switzerland).

### Cytokine detection

TGF-β1 concentrations in the supernatants of TC cells either left untreated or treated with 10 μM FAPi, transfected with a FAP expression vector (Origene, Maryland, USA), siFAP (Thermo Scientific, Bremen, Germany) or mock controls were determined by flow cytometry using a 13-plex human Essential Immune Response Panel a and 1-plex total/free active TGF-β1 detection kit (Biolegend, San Diego, USA) according to the manufacturers’ protocol.

### In vitro FN1 cleavage

For the FN1 cleavage assay, 0.2 µg/µL human recombinant FAP (rFAP; Biolegend) was incubated with 0.1 µg/mL (Biolegend) recombinant FN1 (rFN1) for 1 and 24 hours. The samples were subjected to gel electrophoresis followed by Western blot analysis using an anti-FN1 Ab (company) to assess FN1 cleavage by rFAP.

### Flow cytometry

Single-cell suspensions from cell cultures and co-cultures were stained either for 30 minutes with the apCAF panel consisting of monoclonal antibodies (mAbs) directed against FAP, TEM8, PD-L1, HLA-ABC and HLA-DPDQDR with the myCAF panel consisting of mAbs directed against FAP, TEM8, αSMA, CD9 and CD29 as well as respective isotypes (Table S1). For staining of intracellular markers, cells were fixed and permeabilized using the Intracellular Transcription Factor Buffer Kit (BD Pharmingen) for 20 minutes at 4 °C, followed by washing with Perm/Wash buffer (BD Pharmingen) and subsequent staining with the α-FAP and α-SMA mAbs. The gating strategy for co-culture experiments is provided in Supplementary Figure 2. Flow cytometric analysis was conducted on a CytoFLEX LX instrument (Beckman Coulter, Brea, USA). Data were analyzed using the FlowJo software (Becton Dickinson, New Jersey, USA).

### Immunohistochemical analysis of TC specimen

Conventional IHC was performed on tissue microarrays (TMAs) consisting of 187 primary TC samples of distinct subtypes. The use of the formalin-fixed, paraffin-embedded (FFPE) samples was approved by the Ethical Committee of the Medical Faculty, Martin Luther University Halle-Wittenberg, Germany (2017–81). Two pathologists (U. Siebolts and M. Bauer) independently and blinded to the clinical data scored the stained samples. The antibodies (Ab) used for staining were directed against FAP, pan-HLA class I, HLA-A, HLA-B, PD-L1, CD8 and FN1, respectively (Table S1).

### In silico analysis

Publicly available datasets were analyzed to assess gene expression across TC. Immunohistochemical data from the Human Protein Atlas were evaluated using the online visualization and quantification tools provided by the portal (https://www.proteinatlas.org) for protein expression levels in TC matched normal thyroid tissues as well as across multiple cancer types. Corresponding and RNA expression data were extracted from the TCGA-THCA (https://portal.gdc.cancer.gov/projects/TCGA-THCA) cohort, including tumor (T; *n* = 512) and normal thyroid (N; *n* = 59) samples at the time of the analysis. RNA sequencing (RNA-seq) expression values (FPKM, log2 normalized) were compared between tumor and normal tissues, and associations with clinicopathological parameters and overall survival were analyzed using the GEPIA (http://gepia.cancer-pku.cn) and Protein Atlas platforms. RNA sequencing (RNA-seq) data of conventional cell lines from SANGER database were provided in log2 normalized form and an average of the housekeeping genes actin beta (ACTB), glyceraldehyde-3-phosphate dehydrogenase (GAPDH), ubiquitin C (UBC), 5-aminolevulinate synthase 1 (ALAS1), tubulin beta class I (TUBB), was subtracted from all other genes in each cell line.

The two-tailed Pearson test was employed for the correlation of the Sanger RNA-seq datasets and IHC H-scores between FAP and other molecules. Clinical and histopathological data, for example manifestation of metastasis, were compared to FAP expression levels using the Mann-Whitney U test.

### Statistical analysis

In cell culture experiments, log2 normalization was applied to enhance the visualization and to reduce the fold change between treatments and their respective controls. Unless otherwise stated, the data are presented as means ± standard deviation (SD) from three independent experiments or samples. Statistical analysis was performed using a two-tailed paired Student’s t-test for comparisons between in vitro treatments of biological replicates. Unpaired Student’s t-test was used for IHC H-scores of the same marker between different TC subtypes. A *p* value of < 0.05 was considered statistically significant. Statistical analysis and data visualization were conducted using Microsoft Excel and GraphPad software, respectively.

## Results

### Coordinated upregulated expression of FAP and FN1 in TC

**Fig. 1:**
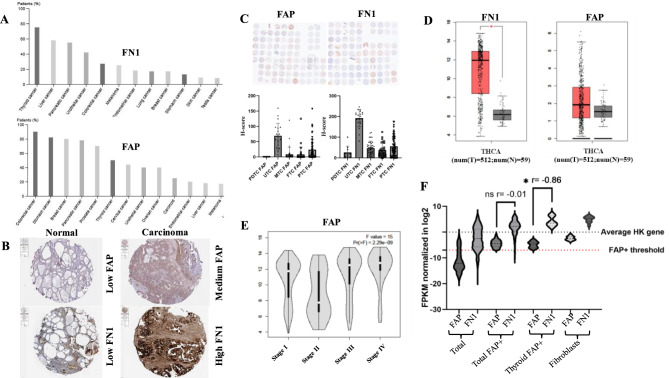
Comparative analysis of FAP and FN1 expression patterns in TC. (**A**) Protein levels of FN1 and FAP across various carcinoma types were extracted from the 3 human protein Atlas raw data repositories showing their differential protein expression pro-4 files. (**B**) Representative histological images of FAP and FN1 of healthy thyroid tissues and 5 TCs obtained from the protein Atlas database. (**C**) Representative overview of FAP and FN1 6 IHC staining of TC TMA (*n* = 187). (**D**) Comparison of the RNA transcription of FN1 and FAP in 7 normal thyroid vs TC using the TCGA dataset. (**E**) Dependence of the FAP mRNA expression 8 on the staging of TC. (**F**) Distribution graph displaying RNA expression levels of FAP and FN1 9 of RNA-seq data obtained from the Sanger database. Pearson correlation analysis was con-10 ducted to evaluate the relationship between FAP and FN1 transcription in the total FAP+ 11 (*p* = 0.389), thyroid FAP+ subclusters (*p* = 0.024) and fibroblasts, respectively. The total number 12 of cell lines included in the RNA-seq dataset was *n* = 1292, tumor cell lines surpassing the FAP 13 threshold *n* = 261, TC cell lines above FAP threshold *n* = 6 and *n* = 29 were classified as fibro-14 blastic cell lines. RNAseq data have been normalized with the average housekeeping gene 15 value set to “0”. (**D**) and (**E**) were directly calculated from GEPIA

Analogous to coordinated high FAP and FN1 expression levels in many cancers [9], in silico analysis of the Human Protein Atlas IHC database demonstrated elevated protein expression levels of both FAP and FN1 in all TC subtypes with the highest FN1 and the 6th highest FAP expression levels in ATC when compared to other cancer types (Fig. 1A), while the expression of FAP and FN1 was low in adjacent non-tumor tissues (Fig. 1B). These in silico data were in line with the IHC analysis of our TMAs composed of 187 differentiated and undifferentiated TC lesions demonstrating significantly increased FN1 and FAP expression levels in TC, in particular in ATC (Fig. 1C).

TCGA analysis of TC cases (T; *n* = 512) and normal thyroid tissues (N; *n* = 59) demonstrated higher RNA expression levels of FN1 and FAP in TC compared to corresponding normal tissues (Fig. 1D), which were correlated with disease progression and worse patients’ outcome (Fig. 1E). Distinguishing FAP^high^ and FAP^low^ expressing tumor cells (Fig. S3A) in TCGA RNA-seq datasets (FAP^high/low^ tumor lesions; *n* = 1292; FAP^high^ carcinoma cell lines; *n* = 237); FAP^high^ TC cell lines; *n* = 6); fibroblastic cell lines (*n* = 29), the highest increase in both FN1 and FAP expression levels was detected in the FAP^high^ TC cell lines mimicking the FAP expression levels of fibroblasts. Interestingly, FAP^high^ TC cell lines revealed a statistically significant negative correlation between FAP and FN1 expression levels (*p* = 0.0121) as demonstrated by Pearson correlation analysis (Fig. 1F).

### FAP-mediated alterations on the cytokine profiles and protein expression

**Fig. 2:**
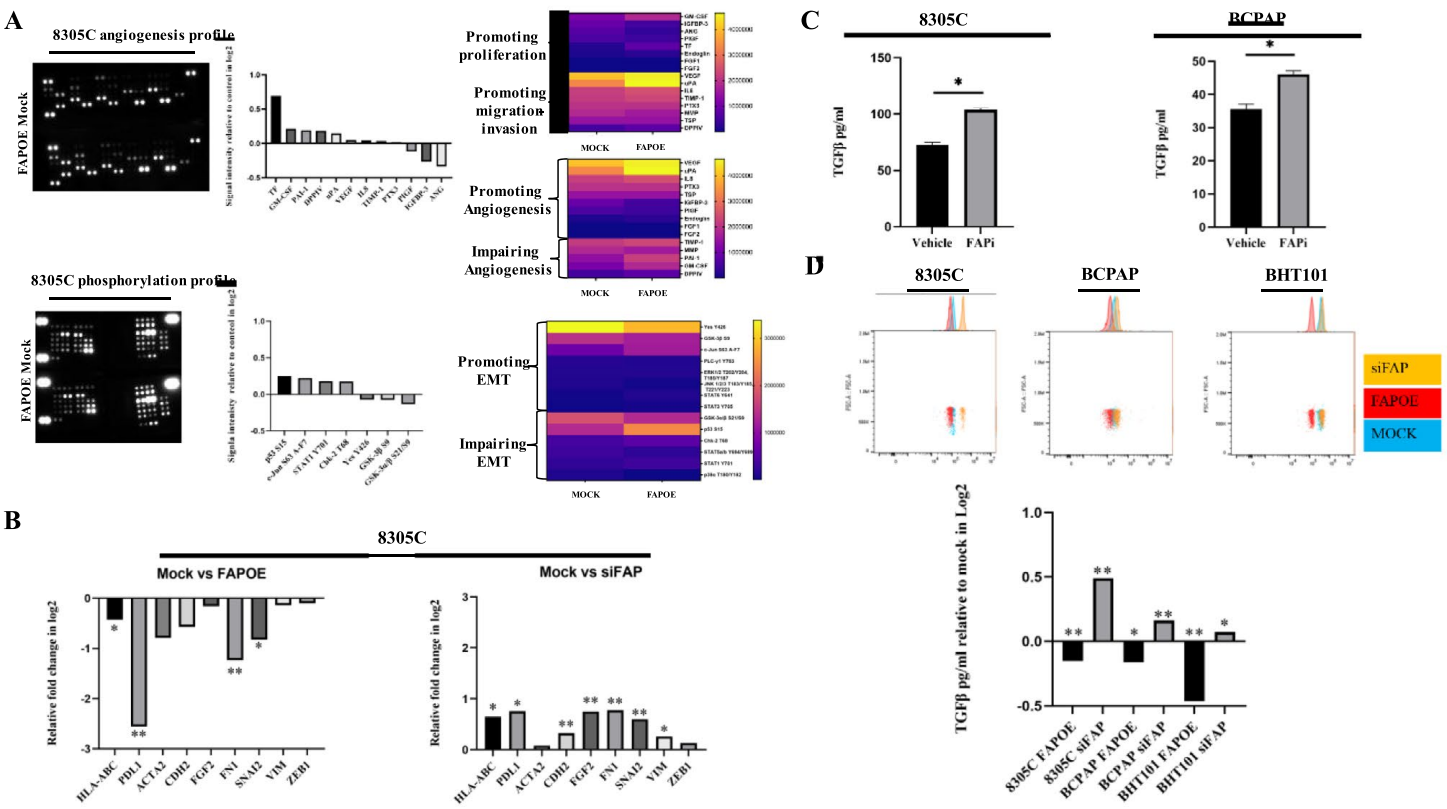
Multifaceted effects of FAP overexpression on angiogenesis, signaling path-20 ways, immune checkpoint regulation and TGF-β activation in TC cells. (**A**) Alterations in the expression of angiogenesis markers and phosphorylation status of proteins 24 in FAPOE TC cell lines vs mock control. Total cell lysates were collected from mock and FAPOE 25 8305 cells 48 hours after transfection and analyzed using two profiler arrays targeting 26 angiogenesis markers and phosphorylated senescence-regulated proteins. After staining with 27 HRP, the HRP signal was quantified for relative expression levels (left) and all target from the 28 profilers were categorized using the STRING database and visualized as heatmap according to 29 their cellular functions and biological characteristics. (**B**) Quantitative qPCR analysis highlights 30 differential mRNA expression of EMT and immune relevant molecules between FAPOE and siFAP-31 transfected 8305C cells. (**C**) Changes in free-active TGF-β levels 48 h after FAP inhibition (10 μM) 32 in the supernatants of 8305C and BCPAP cell lines. A TGF-β-specific ELISA was used to 33 determine the concentration of TGF-β in the supernatants of vehicle and FAPi-treated 8305C (left) 34 and BCPAP (right) cells. The results are presented in pg/ml TGF-β concentration from 3 35 independent experiments. (**D**) Altered TGF-β activation across FAPOE and siFAP-transfected 36 8305C, BCPAP and BHT101 cell lines depicted in log2 ratios relative to mock condition. Data are 37 presented as means of three independent experiments and were analyzed by two tail paired *t*-38 test. *p < 0.05; **p < 0.01

To determine the effect of FAP on the tumor cell phenotype, FAP TC cell lines with known FAP status were generated by FAP overexpression (FAP^OE^) or siRNA-mediated FAP inhibition. In depth analysis of the supernatant of the mock transfected and FAP^OE^ cells using a multiplex cytokine array demonstrated an upregulation of the tissue factor (TF), granulocyte-macrophage colony-stimulating factor (GM-CSF), plasminogen activator inhibitor-1 (PAI-1), dipeptidyl peptidase-IV (DPPIV), urokinase-type plasminogen activator (uPA), vascular endothelial growth factor (VEGF) and interleukin-8 (IL8), but a downregulation of the placental growth factor (PIGF), insulin-like growth factor-binding protein 3 (IGFBP-3) and angiogenin (ANG) (Fig. 2A). Furthermore, the phosphorylation status of selected molecules was determined. Significant alterations in the phosphorylation status of various proteins were detected in mock vs. FAP^OE^ cells, such as e.g., p53 S15, c-Jun S63, STAT1, STAT1 Y701 and Chk-2 T68, while a decreased phosphorylation of YES Y426, GSK-3β S9 and GSK-3α/β S21/S9 was found (Fig. 2A).

### Role of FAP in EMT and immunogenicity of TC

To get further insights into the FAP activity and function in TC cell lines, heatmaps from the profiling data were generated and categorized according to their functional characteristics based on the STRING database. The cytokine profile of FAP^OE^ cells was correlated to invasion/migration vs. proliferation with marginally higher pro-angiogenic characteristics. The phosphorylation status of FAP^OE^ cells demonstrated distinct functions of FAP in the EMT process with decreased expression of markers associated with the promotion of EMT.

An impact of FAP on the expression of immune modulatory and EMT target molecules was demonstrated in Fig. 2Bwith decreased expression levels of HLA-I and PD-L1/CD274 as well as of the EMT markers ACTA2, SNAI2 and FN1 in FAP^OE^ cells. Vice versa, cells transfected with FAP-targeting siRNA resulted in an upregulation of these molecules. Since FN1 is involved in regulating latent TGF-β activation [19, 20], TGF-β levels were determined in the supernatants of TC cell lines in the presence and absence of the FAP inhibitor BR103354 (Fig. 2C). FAP inhibition increased TGF-β levels in the supernatants of 8305C and BCPAP cells, which was confirmed by decreased or increased TGF-β concentrations in the cell supernatants of FAP^OE^ cells and FAP-silenced TC cell lines, respectively (Fig. 2D). To correlate this effect on the expression of immune modulatory molecules, 8305C cells were treated with recombinant TGF-β and analyzed by flow cytometry for the expression of PD-L1, HLA-I and c-MYC. As shown in Fig. S3B, a significant increase of PD-L1 and c-MYC, but a reduced HLA-I expression was found. The effect of FAP inhibition on cellular senescence and cytokine secretion was determined in vehicle and BR103354-treated 8305C cells using apoptosis and cytokine profiler assays (Figs. S3C-D). These data suggest that FAP could promote apoptotic characteristics, which is in line with other studies [21] and favors invasiveness and migratory behavior as recently described [22].

**Fig. 3:**
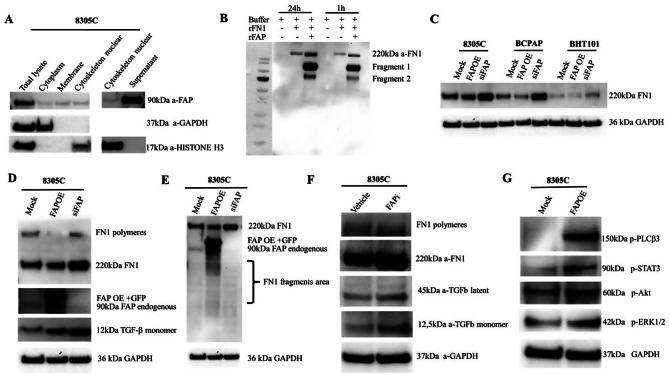
Characterization of intracellular FAP activity and substrate specificity in TC cells. (**A**) Immunoblot of FAP from fractionated lysates and the supernatant of 8305C cells. (**B**) in vitro 43 cleavage assay of rFN1 (100 ng/ml) incubated with rFAP (300 ng/ml). (**C**) FN1 protein expression 44 in three TC cell lines transfected with mock expression vector FAP plasmid and siRNA against 45 FAP. (**D**) Western blot analysis of TGF-β expression in 8305C cells transfected with a mock by 46 FAP expression plasmid and siRNA against FAP. (**E**) Longer exposure time of the membrane 47 showing FN1 fragments in 8305C cells under the same transfection conditions. (**F**) Immunoblot 48 depiction of FAP, TGF-β and FN1 expression in 8305C cells between vehicle (0.1% DMSO) and 49 FAP inhibitor (10 μM). (**G**) Immunoblot depiction of phosphorylated protein targets between mock 50 and FAP overexpressing 8305C cells. All samples with transfection or treatment were collected 51 after 48 h

### FAP localization and activity in TC cells

Based on the diverse effects of FAP on molecules from different compartments, FAP expression was determined in distinct localizations in 8305C cells upon subcellular fractionation. As shown in Fig. 3A, FAP expression was found in the cytoplasm, membrane and nuclear-cytoskeleton, but also extracellularly. Since enzymatic cleavage activities of FAP and FAP-like molecules on the ECM and its link to FN1 alterations have been described [13, 23, 24], the enzymatic activity of FAP was investigated. Indeed, human rFAP cleaved human rFN1 into two fragments indicating its enzymatic activity (Fig. 3B). This interaction was confirmed in the three TC cell lines with distinct FAP status revealing higher FN1 levels upon FAP silencing, but slightly lower FN1 levels upon FAP overexpression (Fig. 3C). This was accompanied by a reduced presence of FN1 polymers, increased FN1 fragments and increased TGF-β levels under both conditions, but particularly upon silencing in 8305C cells (Fig 3D-E). Furthermore, the effect of BR103354 on FN1 and TGF-β levels was comparable to that of siRNA-mediated FAP silencing (Fig. 3F). Elevated p-STAT3, p-ERK1/2 and p-PLCβ3 levels were detected between mock and FAP^OE^ 8305C cells (Fig. 3G), which is in line with the phosphorylation-specific protein arrays and with high levels of the tissue factor (TF) upon FAP^OE^.

**Fig. 4:**
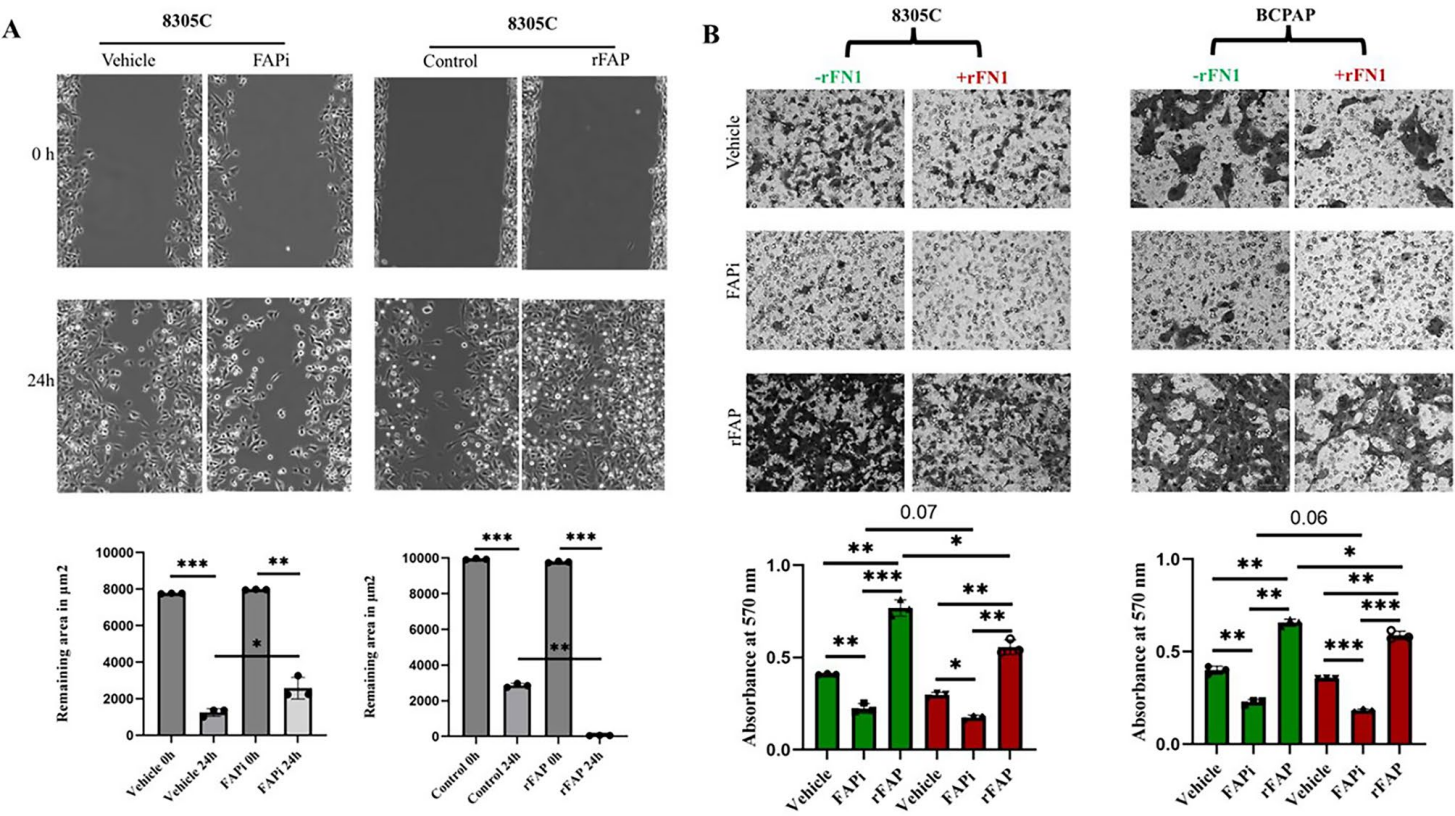
Effects of FAP modulation on wound healing and invasion of TC cells. (**A**) Representative images of a wound healing assay of untreated FAPi- and rFAP-treated 8305 63 C cells. The wound healing assay was performed as described in Materials and Methods and the 64 wound closure was assessed through ImageJ software prior (0h) and 24 hours after treatment 65 with vehicle (0.1% DMSO), FAPi (10 μM) and rFAP (300 ng/ml) in 3 independent experiments 66 (diagram). (**B**) Invasion capacity is evaluated in two TC cell lines via trans-well invasion assays 67 after 24h with representative photos of 8305 C and BCPAP cells depicting invasion with and with-68 out rFN1 (100 ng/ml), and under different treatment conditions: vehicle (0.1% DMSO), FAPi and 69 rFAP (diagram). Quantification of invaded cells was measured via crystal violet staining followed 70 by absorbance measurements at 570nm and depicted in histograms (n=3). Data are presented as 71 mean and SD and were analyzed by two tail paired t-test. *p <0.05; **p <0.01; ***p < 0.001

### FAP-mediated of altered migration and invasion capacity in TC cells

Due to the observed alterations in the angiogenesis and EMT genes (Fig. 2B), the impact of FAP on the mobility dynamics was quantified by wound healing assays of the three TC cells either left untreated or treated with FAPi or rFAP, respectively. As representatively shown for 8305C cells, a statistically significant decrease in migration upon FAP inhibition (*p* = 0.049) was found (Fig. 4A), while treatment with rFAP elicited a pronounced opposite effect (*p* = 0.0002, *n* = 3). The influence of FAP inhibition on cell migration was further evaluated in a time lapse assay confirming the altered migration and morphology (Figs. S4A-B). Comparable results were also shown for three other TC cell lines (Fig. S4C). In addition, FAPi- and rFAP-treated 8305C and BCPAP cell lines demonstrated a reduced invasion upon FAPi treatment (*p* = 0.004) and an augmented invasion independent of rFN1 upon rFAP treatment (*p* = 0.001, *n* = 3) (Fig. 4B). A mitigated invasion between untreated and rFN1-treated conditions were detected in both 8305C (*p* = 0.04) and BCPAP (*p* = 0.04) cells underscoring the complex interplay between FAP- FN1 on the invasion process.

### Morphological changes of spheroids and their FN1 expression upon FAPi treatment

**Fig. 5:**
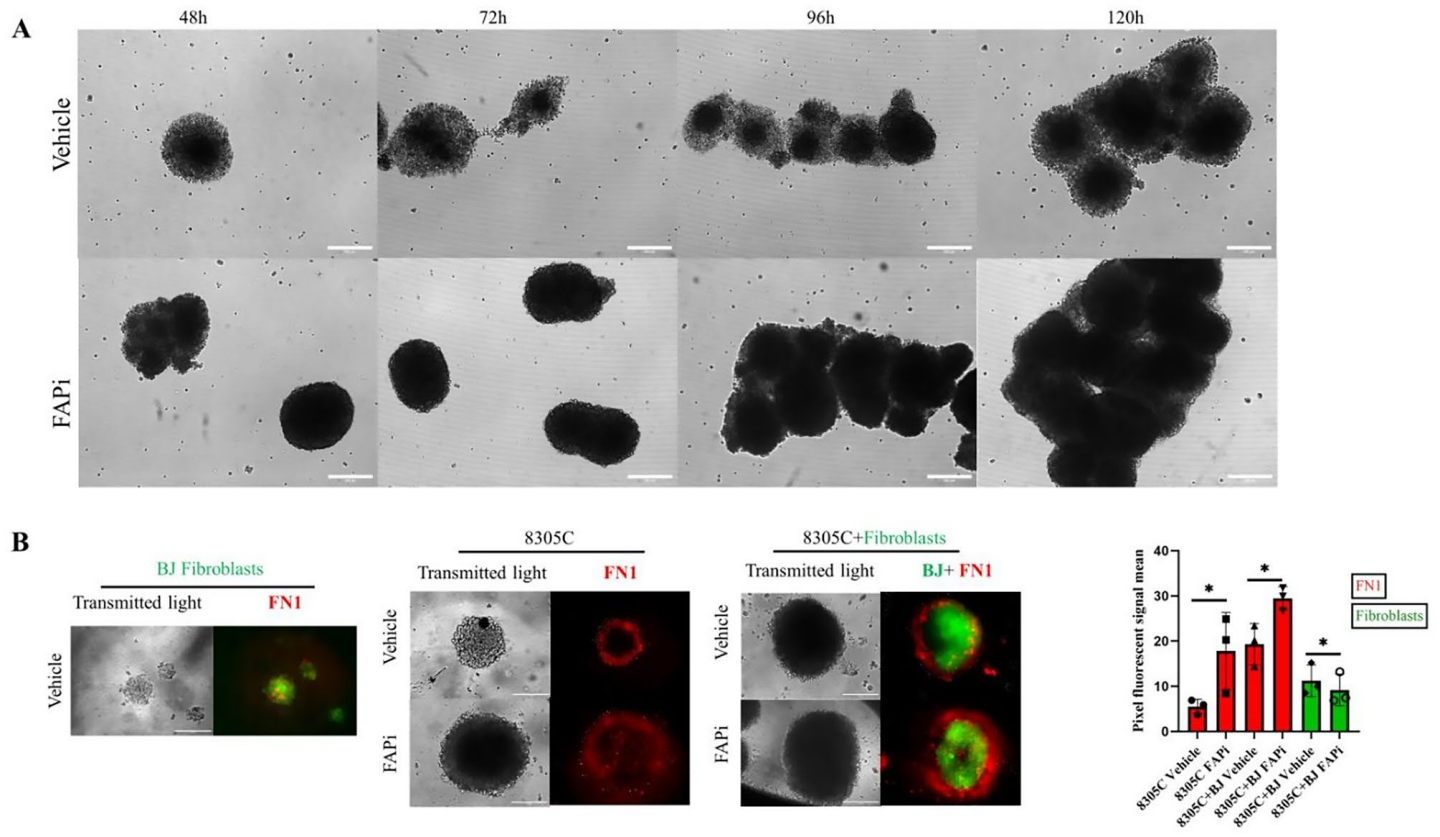
Impact of FAP modulation on spheroid formation and fibroblast interaction in 83 anaplastic TC. (**A**) Representative transmitted light confocal microscopy images of 8305C TC spheroids formed 87 over time (48–120 hours) in the presence of vehicle (0.1% DMSO) and FAPi (10 μM) on low attach-88 ment well plates. (**B**) Immunofluorescence staining of spheroids formed by naive BJ fibroblasts, 89 labeled with CFSE and subsequent staining with an α-FN1 ab conjugated with Zenon Alexa 647 90 in red. Spheroids formed by 8305C TC cells under vehicle (0.1% DMSO) and FAP inhibition 91 (10 μM) conditions are also visualized along with co-culture with BJ fibroblasts (stained with CFSE 92 green) and subsequent α-FN1 ab staining in red (*n* = 3). Images captured after 48 h treatment and 93 ImageJ software used on z-stack image sequences of CFSE green and FN1 red to quantify each 94 respective channel signal and depict them on a histogram plot. Data are presented as mean and 95 SD and were analyzed by two tail paired t-test. *p < 0.05

To get in-depth knowledge of the FAP function and to mimic the in vivo TME, spheroids of 8305C cells were subjected to FAP inhibition for five days and representative confocal images were captured at 24-hour intervals (Fig. 5A). The vehicle-treated spheroids exhibited a relatively sparse morphology with a loose and disorganized morphology in the periphery with evident arrowhead morphology compared to the dense structure of FAPi-treated spheroids supporting a higher invasion rate. To investigate these effects from the ECM perspective, spheroid from vehicle- and FAPi-treated 8305C TC cells in the presence and absence of naïve BJ normal fibroblasts were subsequently analyzed for FN1 expression (Fig. 5B). A statistically significant increased FN1 expression in the presence of FAP inhibition was detected in both mono- (*p* = 0.03) and co-cultures (*p* = 0.01) with comparable BJ fibroblast signals in both co-cultures (*p* = 0.03). In Z-stack projection media files, a higher FN1 expression was detected in the presence of fibroblasts, which might be due to their high basal FN1 expression (Fig. S5). Further insights into FN1 distribution among the slices were obtained by Z-stack imaging and 3D projection animations demonstrating the main location of FN1 at the periphery upon vehicle treatment as already reported for collagen [25], while FAPi treatment resulted in a more ubiquitous FN1 localization around spheroids accompanied by a decreased 2D migratory capacity upon FAP inhibition (Fig. S6). To investigate the enzymatic effects of FAP on FN1, CD29 and HLA-I, untreated and rFAP-treated 8305C TC spheroids were analyzed by confocal imaging (Fig. S7). rFAP treatment caused a more fragmented and less elongated FN1 network, which is in line with the arrowhead morphology (Fig. 5A), while the FN1 and CD29 co-localization remained largely unchanged, but HLA-I expression was slightly elevated in rFAP-treated spheroids. In addition, an increased cell migration was observed with greater distances between nuclei, contrasting with the more organized, dense control structures.

### Alterations of immune modulatory molecules and myofibroblastic markers upon interaction of TC cells with CAFs

**Fig. 6:**
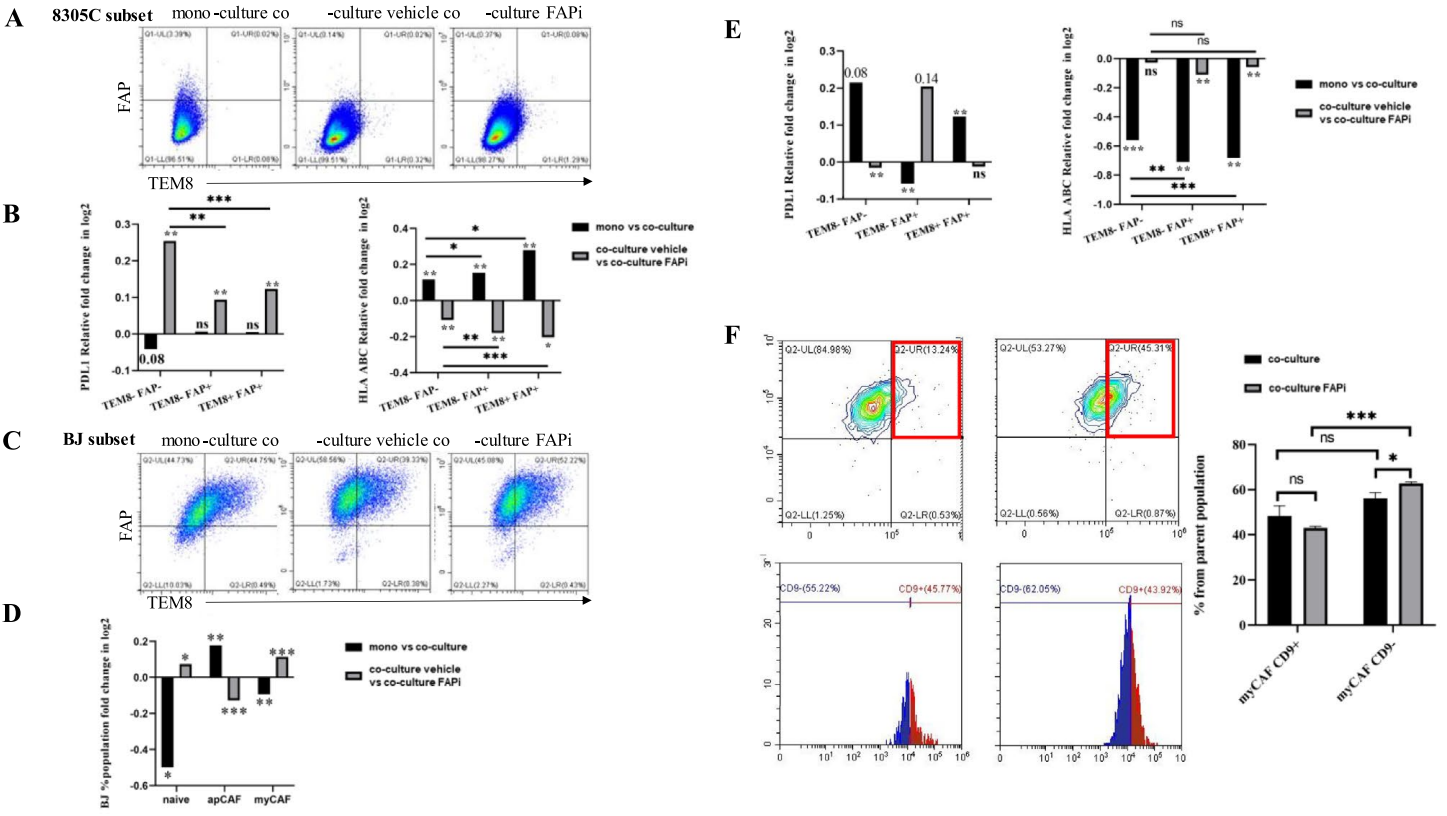
Effect of FAPi on the immune modulatory and myofibroblastic phenotypes of 102 8305C cells, fibroblasts and TC fibroblasts in co-cultures. (**A**) 8305C subset (CFSE-) representative FAP/TEM8 gated dot plots of co-culture experiments 106 (*n* = 3) between ATC cells and fibroblasts with panel 1. (**B**) Subsequent plots of 8305C subset on 107 PD-L1 and HLA-ABC were analyzed on different FAP/TEM8 subpopulations and compared be-108 tween conditions. (**C**) CAF subset (CFSE+) representative FAP/TEM8 gated dot plots of the same 109 co-culture experiments (*n* = 3) between ATC cells and fibroblasts in panel 1. (**D**) Percentile popula-110 tion alterations of apCAF (FAP+, TEM8-) and myCAF (FAP+, TEM8+) were analysed between 111 conditions. (**E**) CAF subset plots analysis of PD-L1 and HLA-ABC expression on different 112 FAP/TEM8 subpopulations and compared between conditions. (**F**) Panel 2 dot plots of CAF sub-113 set, focusing on myCAF (FAP+, TEM8+) subpopulation and sub-gating for CD9± cells, depicting 114%ile population alterations of FAP+TEM8+ CD9+(Wound-/IFNαβ-/acto-myCAF) and CD9- 115 (ECM- and TGFβ-myCAF). All experiments were performed for 48 hours under vehicle (0.1% 116 DMSO) or FAPi (10 μM) conditions (*n* = 3). Panel 1 stained extracellular markers in fresh live cells, 117 panel 2 stained extracellular markers with fresh cells as well as the intracellular markers FAP and 118 αSMA. Data are presented as average ratios in log2 between conditions and were analyzed by 119 two tail paired t-test. *p < 0.05; **p < 0.01, ***p < 0.001

Alterations in the cell surface marker expression due to cell-cell interactions were determined by flow cytometry in co-cultures of 8305C with naïve and CSFE pre-stained fibroblasts using two marker panels representing either the immune modulatory molecules, such as HLA antigens and PD-L1 (panel 1) or the myofibroblastic markers CD29, αSMA, CD9 (panel 2) [26, 27]. For classification of the major CAF clusters, FAP and TEM8 were included as biomarkers. This allowed to categorize CAFs into inflammatory FAP^high^TEM^neg^ iCAF [26, 28], which include antigen-presenting CAFs (apCAFs) and the FAP^high^TEM8^pos^ myofibroblastic CAFs (myCAFs) [29], while naïve CAFs were FAP^low/neg^. 8305C CFSE^-^ cells in mono-culture or co-cultured with BJ fibroblasts CFSE^+^ for 48 hours in the absence and presence of FAPi were stained with Ab panel 1. The 8305C population (Fig. S2) was sub-gated for FAP and then for TEM8 (Fig. 6A) followed by analysis of the PD-L1 and HLA-I expression. A significant increment of PD-L1 expression was detected in FAPi-treated co-cultures, especially in the double negative subset compared to the FAP^+^ TEM8^-^ and FAP^+^ TEM8^+^ subpopulations (*p* = 0.0011; *p* = 0.0006). When compared to fibroblast co-cultures mono-cultures of 8305C cells showed an opposite, but non–significant effect on PD-L1 expression of the FAP^-^ TEM8^-^ subpopulation. A statistically significant induction of HLA-I expression was found when compared mono- to co-cultures with a higher impact on the FAP^+^ TEM8^+^ (*p* = 0.0014) than the FAP^-^ TEM8^-^ (*p* = 0.0228) TC subset. In co-cultures, FAPi treatment reversed the effect on HLA-I with the strongest reduction in the FAP^+^ TEM8^+^ 8305C cells (*p* = 0.019) (Fig. 6B). All markers of Ab panel 1 analyzed on the total TC population demonstrated immune modulatory profile alterations between these culture conditions (Fig. S8A). Further investigations of distinct HLA-I^high/low^ populations showed only a marginal decrement of PD-L1 expression on FAP^-^ /TEM8^-^/HLA-I^low^ vs HLA-I^high^ mono- vs. co-cultured TC cells (Fig. S8B). Analysis of the immune modulatory marker expression on FAP/TEM8 gated CAF subpopulations demonstrated a shift from naïve to apCAF and myCAF subpopulations (Fig. 6C, S8C). Co-culture with TC cells reduced the naïve (*p* = 0.0405) and myCAF population of BJ fibroblasts (*p* = 0.009), respectively, but increased the apCAF population (*p* = 0.0035). This trend was reversed by FAPi treatment resulting in an increased myCAF population (*p* = 0.0001). Co-culture decreased the PD-L1 population in the apCAF subset (*p* = 0.0069), but increased it in myCAF (*p* = 0.0017). In contrast, mono- vs. co-culture of TC on BJ subset showed a significant HLA-I decrease on all three subpopulations with the lowest effect of naïve fibroblasts compared to apCAF and myCAF (*p* = 0.0022, *p* = 0.0004, respectively), while FAPi treatment had an additive effect on this decrease (Fig. 6E). The complete profile between the various culture conditions of TC and BJ cells and their alterations on both panels are depicted in Fig. S8D-E. Sub-gating myCAFs (FAP^+^ TEM8^+^) for CD9^±^ revealed a higher percentage of TGFβ-/ECM-myCAFs (CD9^-^) upon FAP inhibition compared to the vehicle (*p* = 0.0243) as well as a higher percentage of CD9^-^ TGFβ-/ECM-myCAFs compared to CD9^+^ wound-/IFNαβ-/αcto-my-CAFs (*p* = 0.0019) in the presence of FAPi (Fig. 6F). The marker variations of Ab panel 1 described for 8305C cells were also found in the two other TC cell lines (Fig. S9A).

### Correlation of EMT, ECM and immune relevant molecules with clinical parameters in TC

**Fig. 7:**
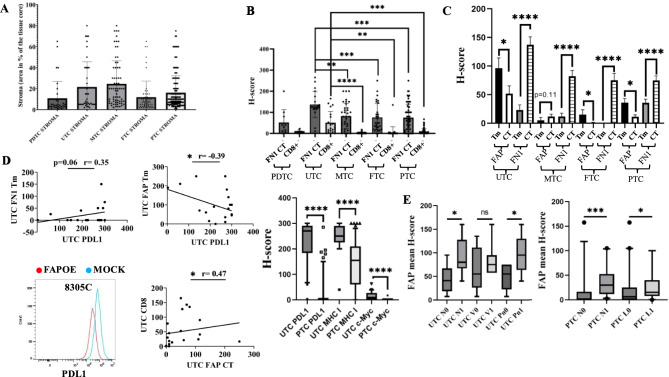
Analysis of stroma composition, immune infiltration and biomarker expression in 121 TC subtypes and correlation with clinical parameters. Various subtypes of TC (n=187; UTC, PDTC, MTC, FTC, PTC) with known clinical data were 125 analyzed by immunohistochemistry (IHC). (**A**) Percentage calculation of stromal tissue across all 126 carcinoma subtypes. (**B**) IHC analysis of FN1 expression in connective tissue and CD8+ T cell 127 infiltration in stromal regions across all subtypes. (**C**) Evaluation of FAP and FN1 expression in 128 tumor and connective tissue with statistical comparison of each target between tumor and 129 connective tissue compartments. (**D**) Pearson correlation analysis of FAP and FN1 expression in 130 tumor tissues versus PD-L1 and CD8 in undifferentiated TC subtype. Histoplot depiction of 8305C 131 transfected cells with FAPOE versus mock on PD-L1 surface expression. Box plot, depicted in 5-132 95 percentile, between UTC and PTC on PD-L1, MHC-I and c-MYC expression patterns. (**E**) 133 Correlation analysis between average H-scores of FAP expression in tumor and connective tissue 134 with clinical metastatic parameters including regional lymph nodes N, blood vessels V, lymph 135 vessel L and perineuronal vessels Pn. Differences in median values between (1) positive and (0) 136 negative samples for N, V, L, Pn criteria are depicted in 5-95 percentile of box plot. Two 137 pathologists independently and blinded to the clinical data scored all samples. 138 Data are depicted as means with SD and (**B**) were analyzed by two tail unpaired t-test, (**C**) by two 139 tail paired t-test, (**D**) were analyzed by two tail paired Pearson correlation as well as two tail 140 8 unpaired t-test for violin plot and (**E**) were assessed using the Mann-Whitney U test. *p < 0.05; 141 **p < 0.01; ***p < 0.001; ****p < 0.0001

To translate the in vitro data into clinical application, a TMA of a TC cohort (*n* = 187 cases) consisting of 20 ATC, 18 PDTC, 84 PTC, 36 MTC and 29 FTC was analyzed by IHC for the expression of key ECM molecules, PD-L1, HLA-I HC and immune cell infiltration. As shown in Fig. 7A, MTC and ATC cases exhibited the highest ratio of tumor stroma versus tumor cells. Based on the significance of FN1 in the ECM formation, its association with stroma and the enzymatic activity of FAP on FN1, the TMA was stained for FAP and FN1 and their expression was evaluated in tumor (Tm) and connective tissues (CT). In addition, the frequency of CD8^+^ T cells was determined in the CT as a marker for stromal immune cell infiltration. The different TC subtypes revealed statistically significant differences in the expression of FN1 CT with higher levels in ATC compared to MTC (*p* = 0.0019), FTC (*p* = 0.0018) and PTC (*p* = 0.0002), respectively. A similar trend was observed for the frequency of CD8^+^ T cell infiltration (Fig. 7B). Higher FAP levels in Tm compared to CT were found for ATC, FTC and PTC with most significant differences in the ATC (*p* = 0.0409). In contrast, an inverse trend was observed for the expression of FN1 in the Tm and CT with the highest statistical significance in ATC (*p* < 0.0001) (Fig. 7C). These data are in line with the negative correlation of FAP and FN1 of the RNA-seq data (Fig. 1F). To correlate the FAP and FN1 to the expression of immune response relevant molecules, the TMA was further stained with anti-PD-L1 and two anti-HLA-I Ab (Fig. 7D). In ATC, a statistically significant negative correlation was found between FAP and PD-L1 (*p* = 0.0423), but a non-statistically significant (*p* = 0.06) positive association on FN1 and PD-L1, which becomes statistically significant (*p* = 0.0113) using the total TC cohort (Fig. S9B). Concerning the link of CD8^+^ T cell infiltration and HLA-I, a non-statistically significant trend of a positive correlation between CD8^+^ T cells and HLA-I was found in ATC (*p* = 0.297), which became significant on the total TC cohort (*p* < 0.0001) (Fig. S9B). The inverse correlation of PD-L1 with FAP was in line with our results on two TC cell lines transfected with FAP^OE^, where PD-L1 was significantly decreased (Fig. 7D; Fig. S9C). In agreement with the FAP-mediated reduction of the ECM integrity, a strong positive correlation between FAP and CD8^+^ T cells was found on tumor stroma (*p* = 0.0329). Furthermore, PD-L1 (*p* < 0.0001), MHC-I (*p* < 0.0001) and c-MYC (*p* < 0.0001) expression was statistically significant higher in UTC versus PTC (Fig. 7D).

The FAP staining correlated to the tumor stage and disease progression with a significant association of FAP expression levels in the Tm and CT compared to the metastatic spread in regional lymph nodes N (*p* = 0.0225) and perineural invasion Pn (*p* = 0.017) of anaplastic UTC (Fig. 7E) suggesting an increased tumor cell dissemination to regional lymph nodes and perineuronal vessels with elevated FAP expression. In addition, significant correlations were detected in PTC for the lymph node status N (*p* = 0.00014) and lymphatic vessel L (*p* = 0.0367) underscoring the presence of FAP in promoting metastatic spread to lymph nodes and blood vessels.

## Discussion

**Fig. 8:**
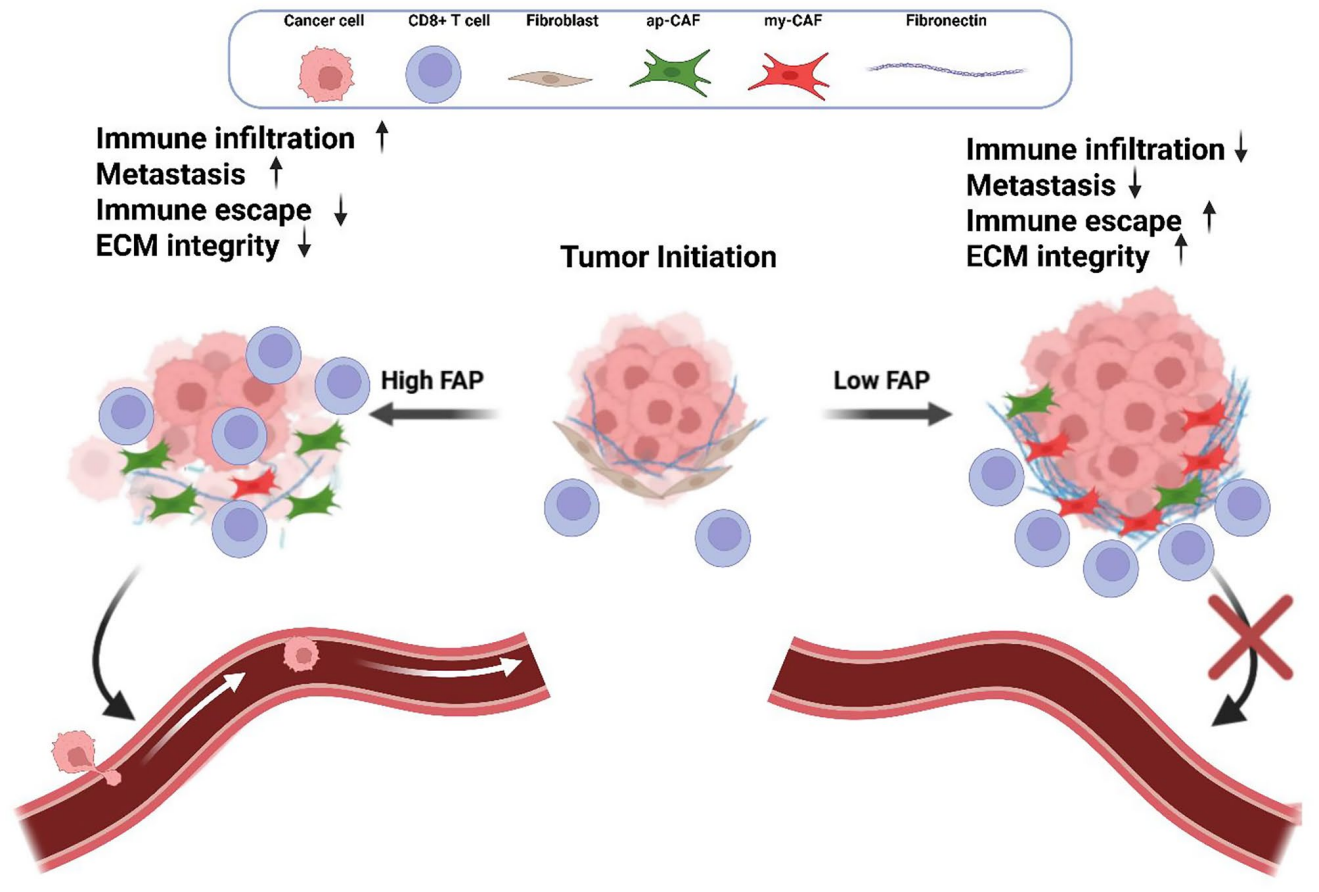
Putative role of FAP on tumor immunogenicity and metastasis of TC. High levels of FAP in TC promote metastases and alter the immune landscape by cleaving FN1 184 and subsequently lowering TGF-β activation resulting in reduced PDL1 and increment of MHC 185 class I expression. In addition, the frequency of my-CAF subpopulation is also lowered 186 accompanied by a reduced ECM integrity and an increased CD8+ T cell infiltration

To the best of our knowledge, this study delineates for the first time the intricate dynamics of FAP and its regulatory effects on TC progression determined by changes of the EMT markers and induction of immune escape with a complex interplay between cellular proliferation and migration-invasion mechanisms (Fig. 8). The role of FAP extends beyond molecular interactions influencing broader tumor behaviors and microenvironmental interactions by its enzymatic cleavage of FN1, which affects the availability and activation of TGF-β. FAP directly modulates FN1 integrity and its interaction with CD29, which is crucial for activating latent TGF-β known to be involved in differentiation, migration and immune evasion [30–32]. The altered FN1 polymerization by FAP, not only changes the structural composition of the ECM, but also significantly influences the TGF-β signaling pathway resulting in lower EMT marker expression. In tumor progression, a time-dependent trade-off between cell proliferation and migration has been observed with tumors favoring migration under certain conditions [33–35]. The angiogenesis profile characterized in this report showed a higher preference for migration and invasion rather than for proliferation upon FAP overexpression. This is supported by the cytokine profile upon FAP inhibition highlighting the role of FAP in tumor cell behavior and TME, which is enhanced by the activation of the MAPK pathway via the TGF - PARP1/2 - PLCβ axis, known to be regulated by the phosphorylation of PLCs via G coupled protein activation [36] accompanied by higher uPA, VEGF and IL8 levels [37, 38]. In addition, this is in line with a reduced migration and invasion in TC cells upon FAPi treatment, while rFAP administration had the opposite effect. These findings were consistent using 2D and 3D in vitro models. FAP inhibition results in a more compact and organized structure of spheroids, which contrasts the unrestrained and more dispersed morphology in the presence of rFAP. The structural variance not only supports the role of FAP in the cellular arrangement and ECM interaction, but also in the migratory and invasive capacity of cells [39–41]. Flow cytometric analysis of co-cultured TC cells and CAF treated with FAPi revealed higher PD-L1 levels in TC cells suggesting FAP as negative regulator of PD-L1, while it enhances the HLA-I and HLA-II expression. This can be explained by the TGF-β-mediated upregulation of PD-L1 and downregulation of HLA-I in tumor models as shown by us and various reports [42–48] suggesting the involvement of FAP in shaping the immune-suppressive TME landscape. Furthermore, FAP inhibition resulted in a strong immune-evasive phenotype of the co-cultured CAFs by increasing the frequency of myCAFs and lowering the number of apCAFs, which has a significant impact on the activation and infiltration of immune cells into the tumor [49–51]. This FAP-driven phenotype highlights its dual role in both tumor progression and immune evasion. Inhibition of FAP increased the immune-evasive features of TC cells and CAFs associated with characteristics resembling a “cold” tumor but reducing the motility indicating a potential therapeutic favorable exchange. Additional complexity was noted by sub-gating the myCAF subpopulations using CD9 as marker, a tetraspanin family member, which is facilitating the association and organizing other functional proteins like CD29 and TGFBR1 [52–54]. Next to the myofibroblastic characteristics with increased levels of CD29 and αSMA upon FAP inhibition, a differential increase of ECM-myCAFs underscores the nuanced roles that these subpopulations play within the tumor stroma. They influence the structural integrity and TGF-β signalling as well as modulate the immune landscape in tumors, which is in line with the simultaneous higher levels of PD-L1 responsible for the reduced frequency of tumor infiltrating lymphocytes (TILs) [27, 55–57].

Immunohistochemical staining of TC-specific TMAs highlighted the differential expression patterns of FAP and FN1 across various TC subtypes with a particularly high FAP expression in more aggressive TC. Further analysis revealed a trend between connective tissue and tumor area, where FAP has a reverse relation with FN1 on tissue location, which is in accordance to the negative correlation between FAP and FN1 in TCGA seq data on FAP^high^ TC cells. Accumulated CD8^+^ TILs in the stroma positively correlated with FAP presence strengthening our hypothesis that alterations of the ECM-, wound-myCAF ratio by FAP levels, could lead to a reduced stroma integrity and consequently an increase of TILs. A significantly higher PD-L1 and c-MYC expression was found in aggressive undifferentiated TC compared to the well differentiated counterparts. Additionally, a comprehensive analysis on aggressive TC depicted a comparable negative correlation trend between FAP and PD-L1 and a positive between FN1 and PD-L1, which was in accordance with our in vitro findings of TGF-β treatment and FAP^OE^ transfection with an increase of PD-L1 and c-MYC, but a decrease of PD-L1 in the TC cells, respectively. Furthermore, significant positive correlations of FAP with clinical metastatic markers in regional lymph nodes and perineural invasion exists thereby strengthening our hypothesis.

## Conclusions

This study underscores the pivotal role of FAP in driving TC progression, specifically by enhancing tumor cell migration, invasion and immune cell infiltration, while regulating PD-L1, HLA-I and my-CAF subpopulations. Beyond its powerful prognostic value, FAP also serves to distinguish between TC subtypes. The dual actions of FAP mediated by its regulation of FN1-TGF-β signaling axis, highlight a unique therapeutic opportunity. FAP inhibition may initially accelerate tumor growth, creating a larger, but less invasive tumor that is more accessible for surgical removal. This approach, especially when combined with TGF-β inhibitors and immunotherapies like anti-PD-L1 antibodies or CAR T cell therapy, could shift the disease trajectory, offering patients with aggressive TC an effective strategy to control metastasis and enhance surgical outcomes.

## Electronic supplementary material

Below is the link to the electronic supplementary material.

Supplementary Material 1

Supplementary Material 2

Supplementary Material 3

Supplementary Material 4

Supplementary Material 5

Supplementary Material 6

Supplementary Material 7

Supplementary Material 8

Supplementary Material 9

Supplementary Material 10

Supplementary Material 11

Supplementary Material 12

Supplementary Material 13

## Data Availability

All data associated with this study are present in the paper or the Supplementary Materials. The full clinical data, IHC scores and other raw values, are uploaded in Figshare server 10.6084/m9.figshare.26147350. They can be requested by academic researchers and will be provided after review and approval of a research proposal under Data Sharing Agreement (DSA).

## Abbreviation

Ab: Antibody
ACTA2/ΑAsma: Alpha smooth muscle actin
ACTB: Actin beta
ALAS1 5: Aminolevulinate synthase 1
ANG: Angiogenin
ATC: Anaplastic thyroid carcinoma
ATCC: American Tissue Culture Collection
BSA: Bovine serum albumin
CAF: Cancer-associated fibroblast
CFSE: Carboxyfluorescein succinimidyl ester
DAPI: 4’,6-Diamidino-2-phenylindole
DMSO: Dimethyl sulfoxide
DPPIV: Dipeptidyl peptidase-IV
ECL: Enhanced chemiluminescence
ECM: Extracellular matrix
EDTA: Ethylenediaminetetraacetic acid
EMEM: Eagle’s modified essential medium
EMT: Epithelial-to-mesenchymal transition
ERK1/2: Extracellular signal-regulated kinase 1/2
FAP: Fibroblast activation protein
FAPi: FAP inhibitor
FAP^OE^: FAP-overexpressing
FBS: Fetal bovine serum
FCS: Fetal calf serum
FFPE: Formalin-fixed paraffin-embedded
FGF1/2: Fibroblast growth factor 1/2
FN1: Fibronectin 1
FPKM: Fragments per kilobase of transcript per million mapped reads
FTC: Follicular thyroid carcinoma
GAPDH: Glyceraldehyde-3-phosphate dehydrogenase
GFP: Green fluorescent protein
HLA-I: Human leukocyte antigen class I
GM-CSF: Granulocyte-macrophage stimulatory factor
HRP: Horseradish peroxidase
ICP: Immune checkpoint
IGFBP-3: Insulin-like growth factor-binding protein 3
IHC: Immunohistochemistry
IL8: Interleukin 8
MHC: Major histocompatibility complex
MTC: Medullary thyroid carcinoma
p38α: p38 alpha mitogen-activated protein kinase
p53: Tumor protein p53
PAI-1: Plasminogen activator inhibitor 1
PBS: Phosphate buffered saline
PDGFRβ: Platelet-derived growth factor receptor beta
PD-L1: Programmed death ligand 1
PDTC: Poorly differentiated thyroid carcinoma
PIGF: Placental growth factor
PLC-γ: Phospholipase C-gamma 1
PTC: Papillary thyroid carcinoma
PVDF: Polyvinylidene difluoride
rFAP: Recombinant FAP
RNA-seq: RNA sequencing
SD: Standard deviation
siRNA: Small interfering ribonucleic acid
SNAI2: Snail family transcriptional repressor 2
STAT1: Signal transducer and activator of transcription 1
STAT2: Signal transducer and activator of transcription 2
STAT: Signal transducer and activator of transcription
TC: Thyroid carcinoma
TCGA: The Cancer Genome Atlas
TEM8: Tumor endothelial marker 8
TF: Tissue factor
TGF-β: Transforming growth factor beta
TMA: Tissue microarray
TME: Tumor microenvironment
TUBB: Tubulin beta class I
UBC: Ubiquitin C
uPA: urokinase-type plasminogen activator
VEGF: ascular endothelial growth factor
