# Supplementary material for: Fibroblast activation protein (FAP)-mediated promotion of metastasis via the FN1-TGFβ axis and immune suppression in aggressive thyroid cancer
Source: J Transl Med. 2025 Nov 13;23:1284. doi: 10.1186/s12967-025-07307-3 (PMC12616960; doi:10.1186/s12967-025-07307-3)
Supplement: Supplementary file 1 — Supplementary Material 1 [file 12967_2025_7307_MOESM1_ESM.pdf]

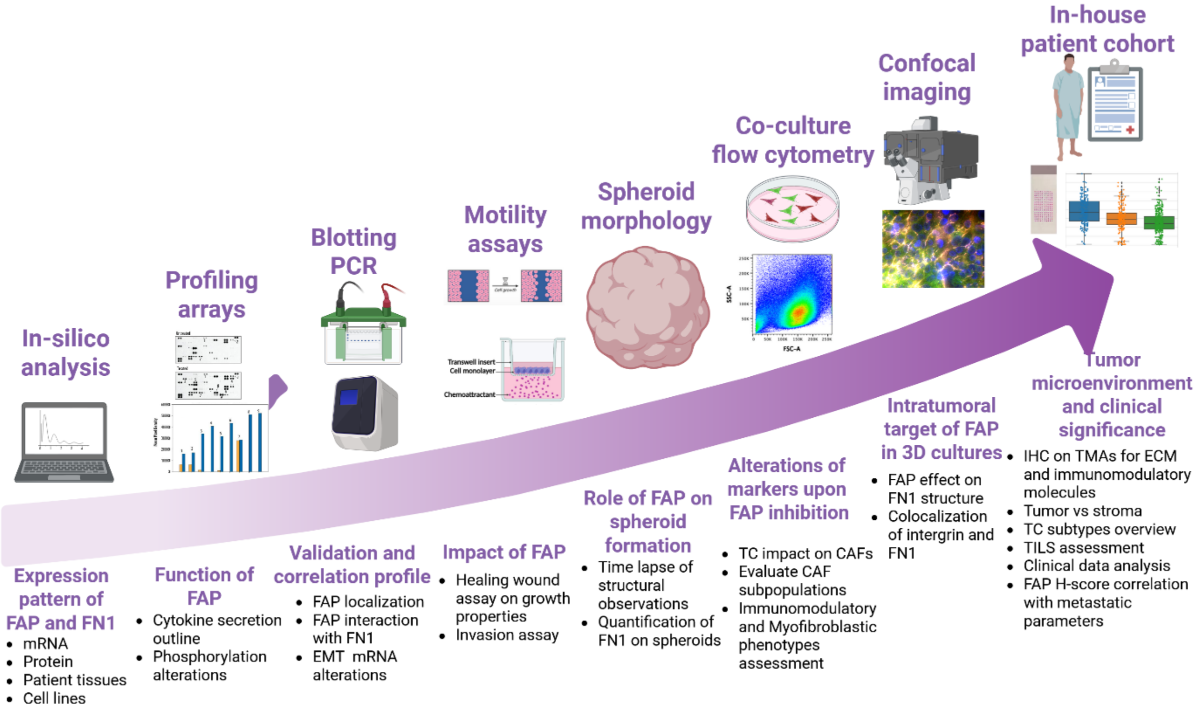

S1. Workflow overview

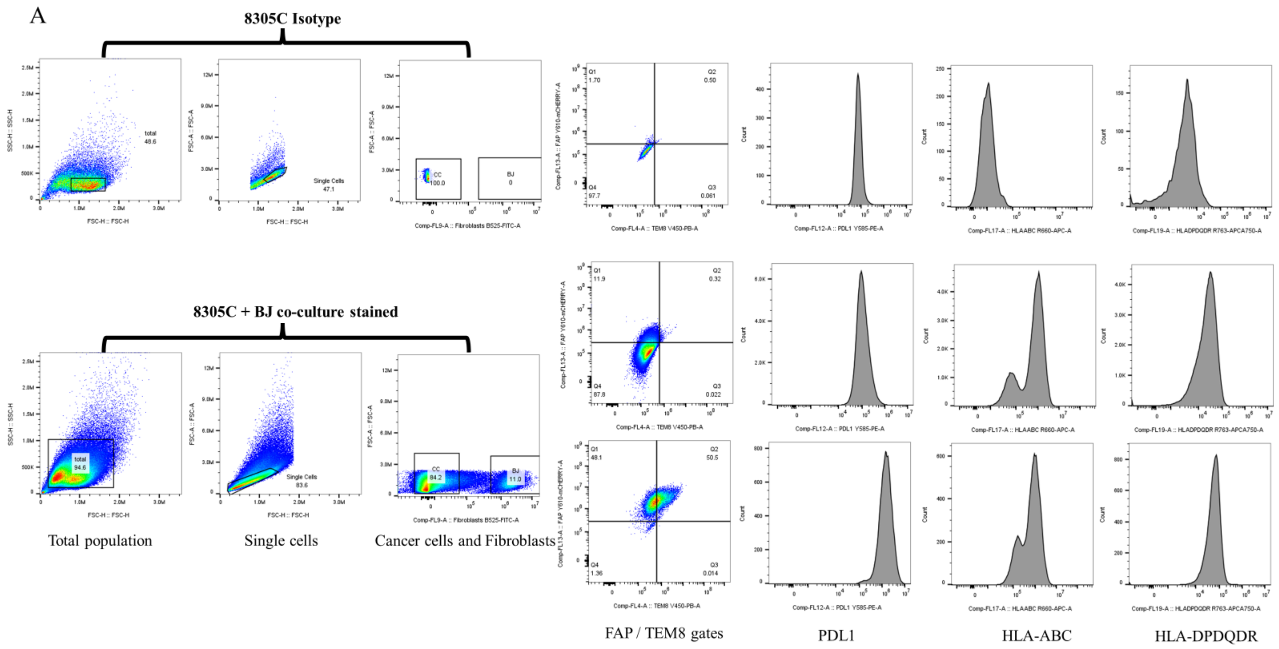

S2. (A) Flow cytometry gating example between isotypes of 8305C and 8305C+BJ fibroblasts stained for panel 1, extracellularly stained (FAP-alexa594, TEM8-alexa405, PDL1-PE, HLA-ABC-APC, HLA-DPDQDR-APCCy7). BJ fibroblasts were stained with CFSE prior to the assay and gated under FITC fluorescent channel.

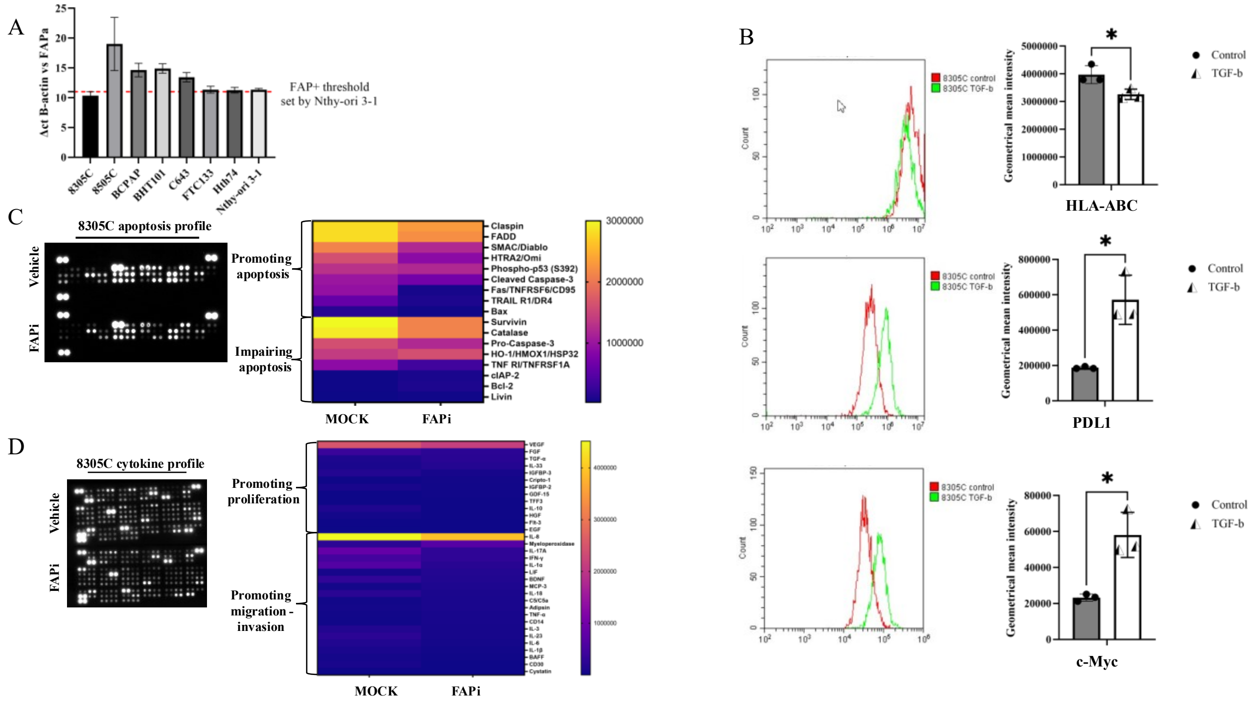

S3. (A) Analysis of mRNA FAP expression in TC cell lines and one immortalized normal thyroid cell line on data on A are presented as Δct mean with SD using RT-PCR. The results are depicted in Δct between housekeeping gene and FAP, for threshold set on FAP high (FAP+). (B) 8305C cells were treated with recombinant 400ng/ml TGF-β for 72 hours before PDL1, HLA-ABC and c-myc expression was determined by flow cytometry. (C) Proteome profiler blots of total lysate of TC cells treated for 48 hours with vehicle (0.1% DMSO) or FAPi (10μM). Alterations in cellular apoptosis markers depicted in HRP signal between vehicle and treatment are shown in a heatmap. (D) Proteome profiler blots of supernatant, comparing vehicle (0.1% DMSO) and FAPi (10μM) after 48h shows alterations in angiogenesis markers depicted in HRP signal between vehicle and treatment in a categorized heatmap for proliferative and migratory effects. Data on B are presented as geometrical mean with SD and were analyzed by two tail paired t-test. *P < 0.05

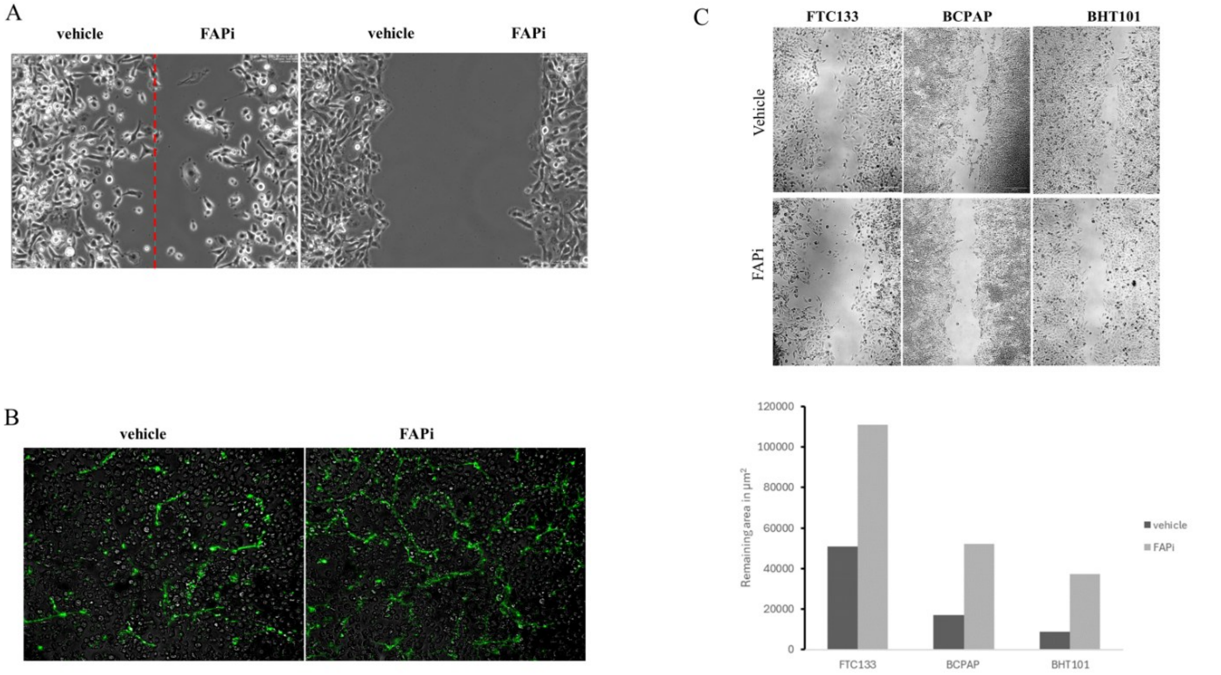

S4. (A) Last frame of a video time-lapse between vehicle (0.1% DMSO) and FAPi (10μM) 24h with a red line defining the center of the image (left) and the total real time video of the same experiment (right). (B) Staining for FN1 live cells conjugated with alexa488 of the same experiment. (C) Wound healing effect (n=1) in 3 other TC cell lines. The covered area was assessed through imageJ software and depicted in μm^2 remaining area histograms of 0h and 24 hours comparing vehicle (0.1% DMSO) and FAPi (10μM) treatment.

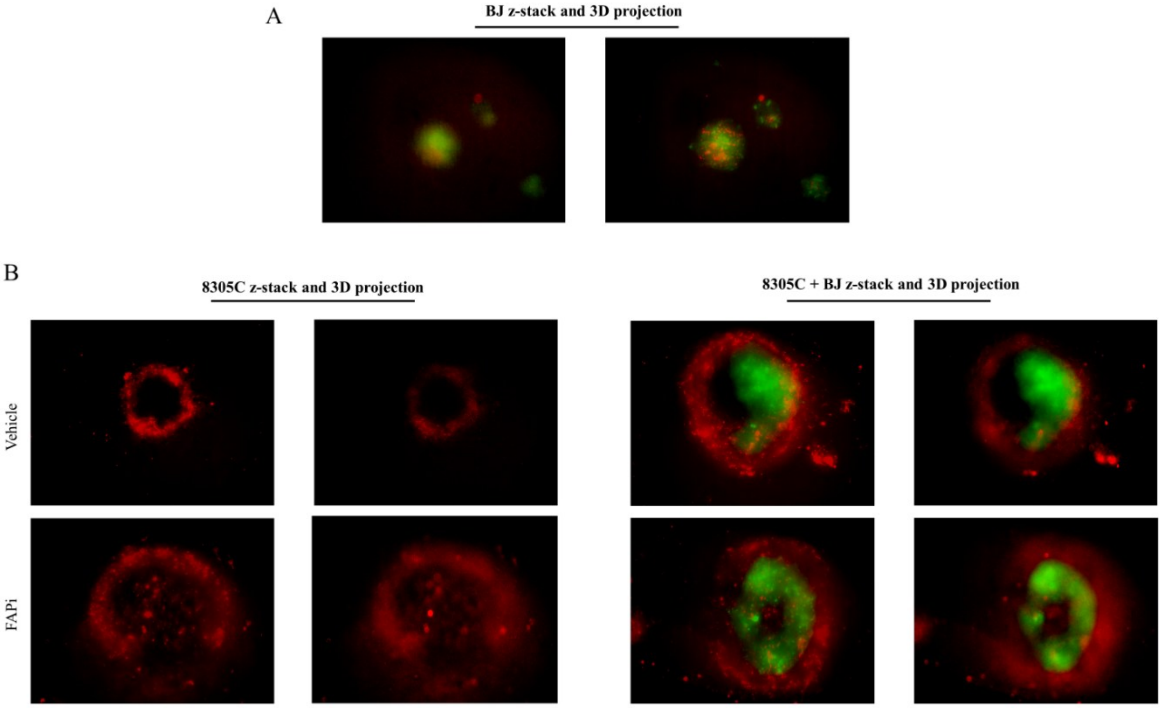

S5. Media gif files of figure 5B. z-stack and 3D projection media files of (A) mono-culture of BJ fibroblasts and (B) mono-culture culture of 8305C and co-culture of 8305C and BJ between vehicle and FAPi.

A

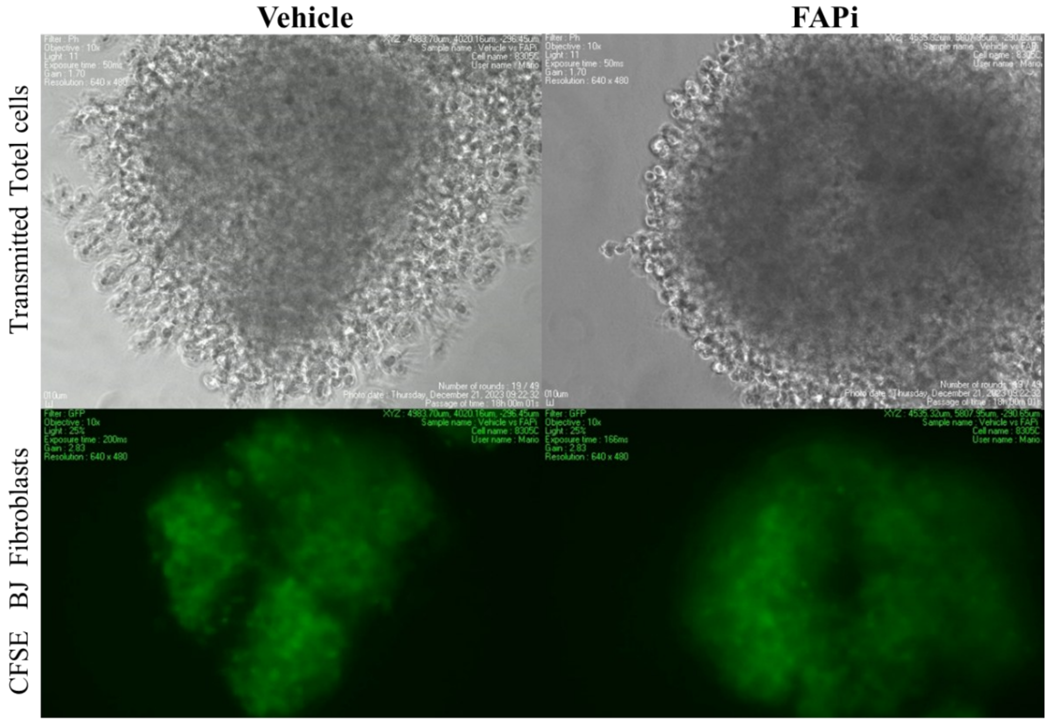

S6. (A) Real time video file of 8305C+BJ fibroblasts co-cultured in low attachment plates to form spheroids followed by proliferation in normal well plates between vehicle (0.1% DMSO) and FAP (10μM) conditions. Prior to the assay fibroblasts were stained with CFSE and visualized under Biostation IM-Q real-time GFP fluorescent system

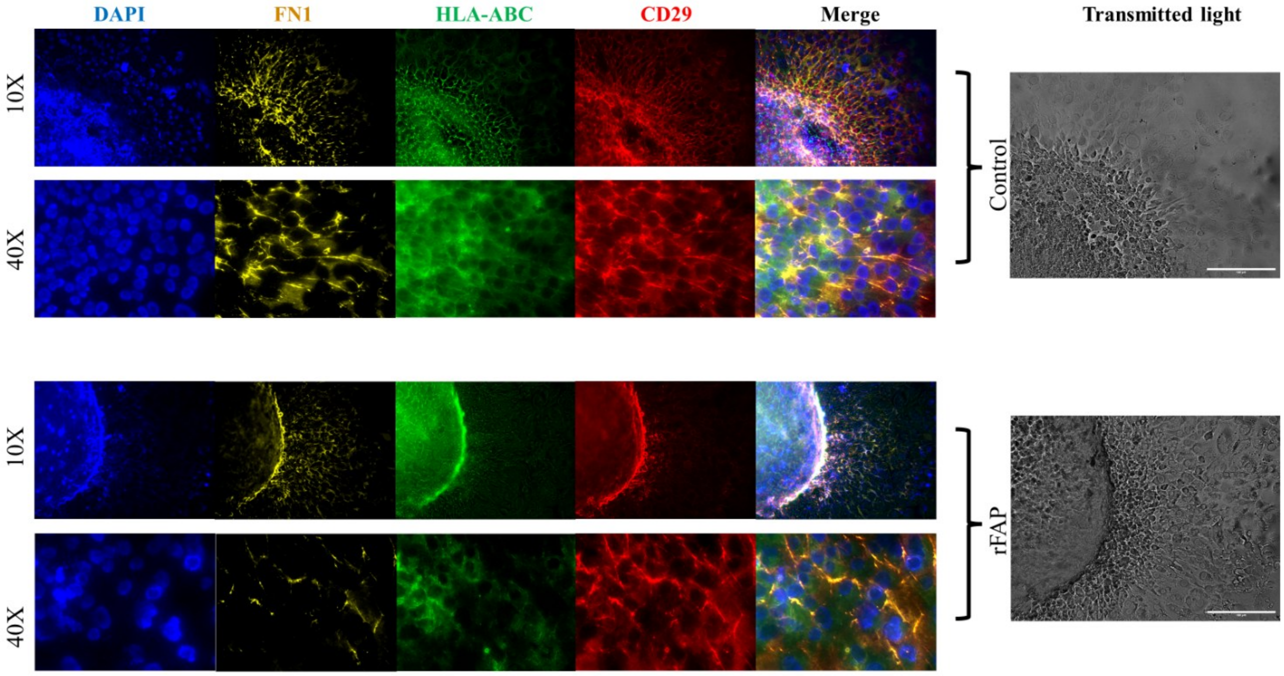

S7. Representative images (n=3) from spheroids formed by 8305C cells in low attachment well plates were allowed to proliferate in normal well plates between control and rFAP (300 ng/ml). After 24 hours, confocal imaging was captured showing five channels: CD29 integrin β1 PE (red), HLA-ABC FITC (green), FN1 conjugated with Alexa 647 (yellow), DAPI (blue) and merged images alongside transmitted light. The images captured at both 10x and 40x magnification reveal the localization and morphology of FN1 structures and their relationship with migrated cells from the main spheroid. The individual channel images as well as the transmitted and merge were separated and defined signal color using imageJ software.

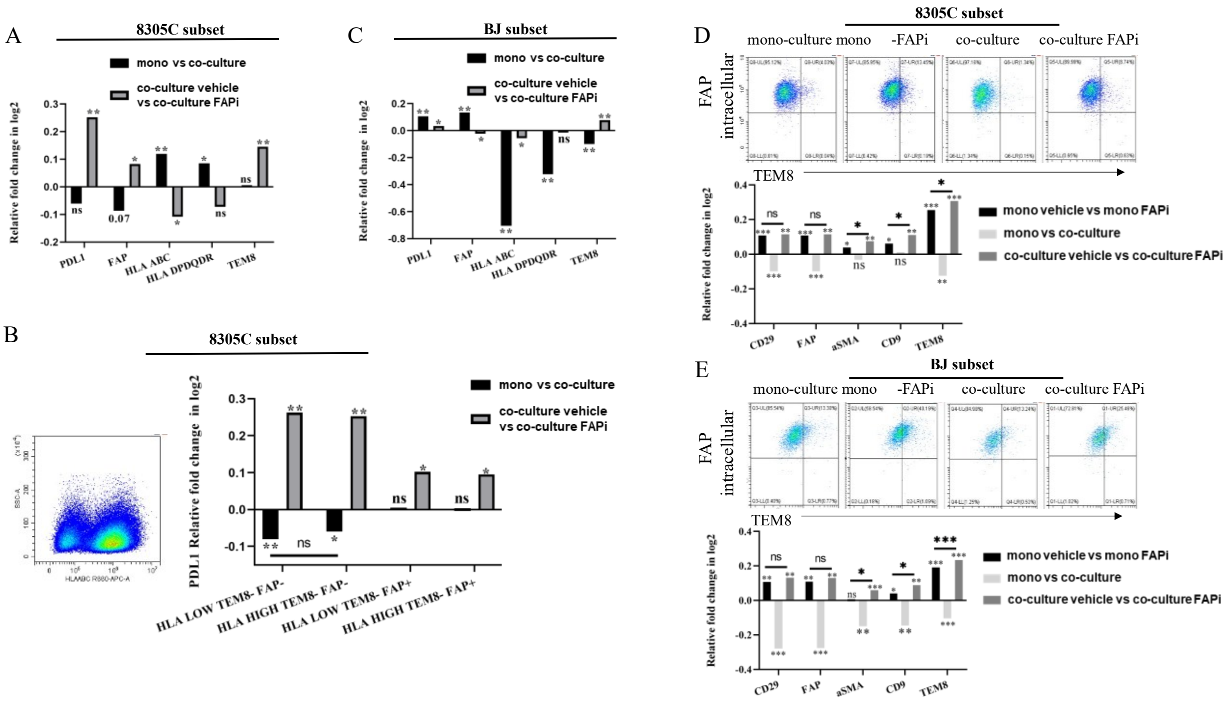

S8. (A) Supplementary 8305C flow cytometry data of 8305C cells stained with Ab of panel 1 in total population using the conditions shown in Figure 6A. (B) PDL1 expression on HLA-ABC$^{high/low}$ population and FAP-TEM8 subsets on cancer cells between conditions. (C) Supplementary flow cytometry data of BJ cells stained with Ab of panel 1 in total population between conditions from Figure 6C. (D) Flow cytometric data of 8305C panel 2 in total population and (E) BJ panel 2 targets in total population between conditions with intracellular FAP staining. Data are presented as log2 means and were analyzed by two tail paired t-test. *P < 0.05, *P < 0.01, *P < 0.001

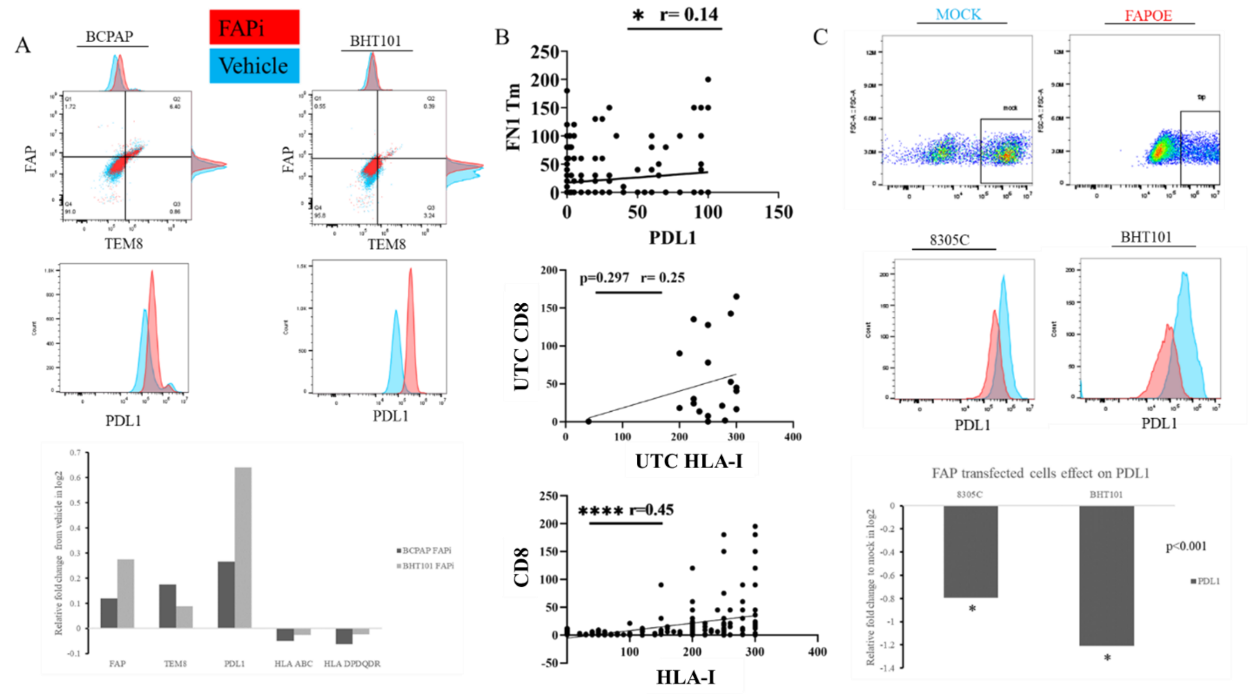

S9. (A) Flow cytometry on 2 other TC cell lines (n=1), single culture with no fibroblasts, stained with Ab of panel 1 and with same condition as Fig.6A. Histogram depiction of all targets as well as PDL1 effect on FACS histoplots. (B) Total TC subtype correlation between FN1 Tm and PDL1, as well as UTC and total TC subtype correlation between CD8 and HLA-I. (C) 8305C and BCPAP cells were transfected with mock (mCherry) and FAP overexpression (GFP) plasmid (n=3). After 48h cells were stained for PDL1 and measured with flow cytometry after gating for positive transfected cells. Data from A and C are presented as log2 means and were analyzed by two tail paired t-test, while results from B were analyzed by two tail unpaired Pearson correlation *P < 0.05 ****P < 0.0001
