# Supplementary figures and images for: Fibroblast activation protein (FAP)-mediated promotion of metastasis via the FN1-TGFβ axis and immune suppression in aggressive thyroid cancer
Source: J Transl Med. 2025 Nov 13;23:1284. doi: 10.1186/s12967-025-07307-3 (PMC12616960; doi:10.1186/s12967-025-07307-3)

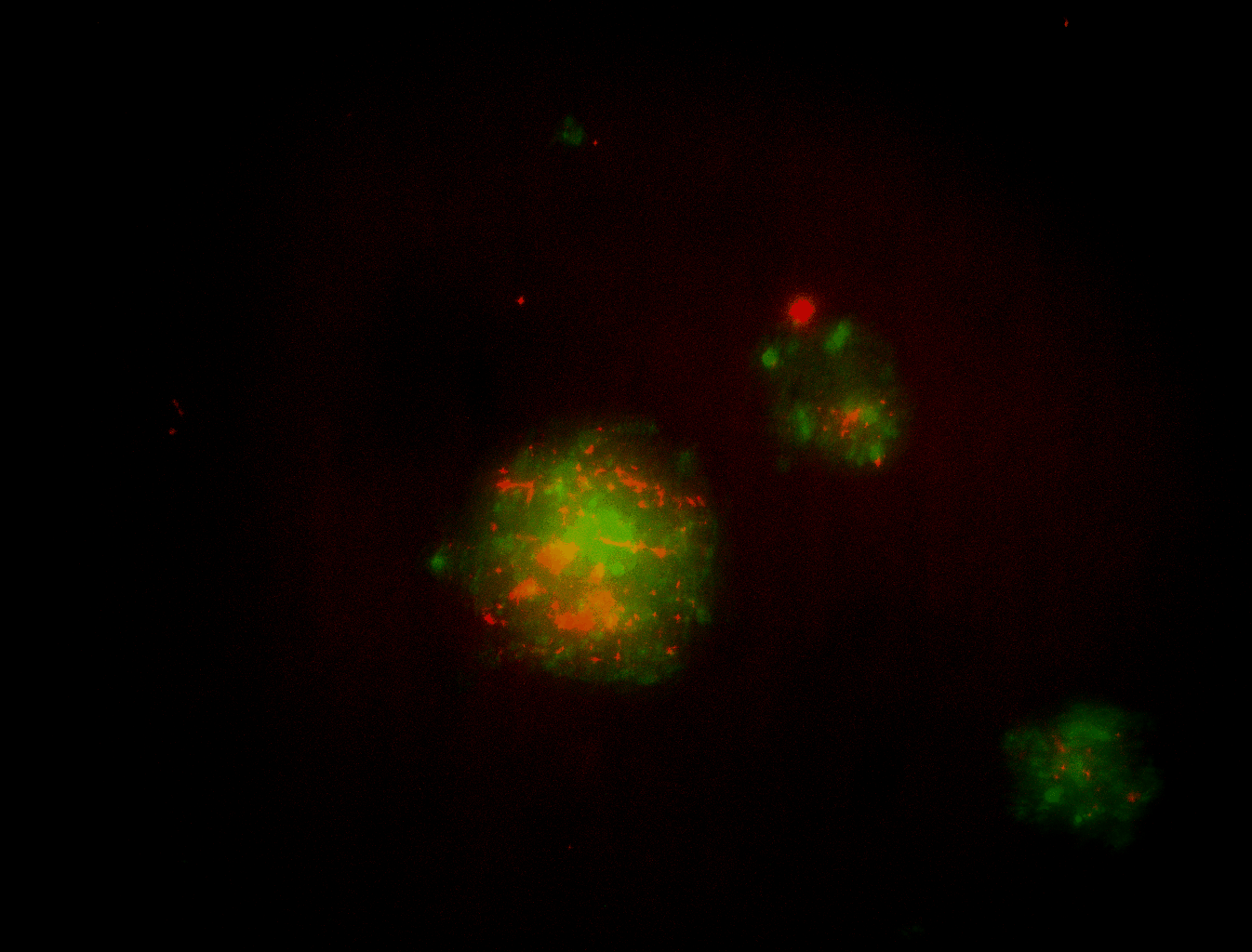

Supplement: Supplementary file 2 — Supplementary Material 2 [file 12967_2025_7307_MOESM2_ESM.gif]

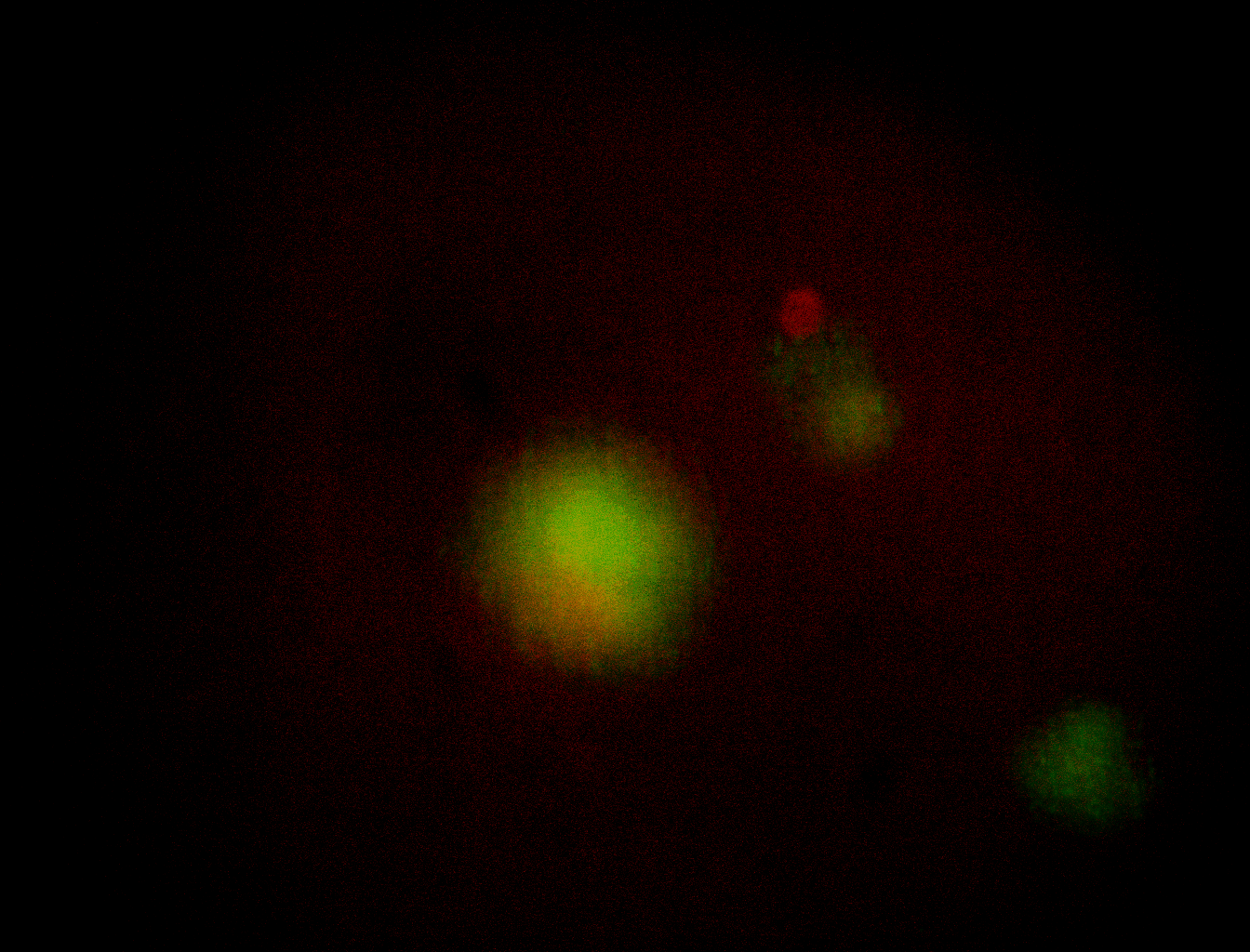

Supplement: Supplementary file 3 — Supplementary Material 3 [file 12967_2025_7307_MOESM3_ESM.gif]

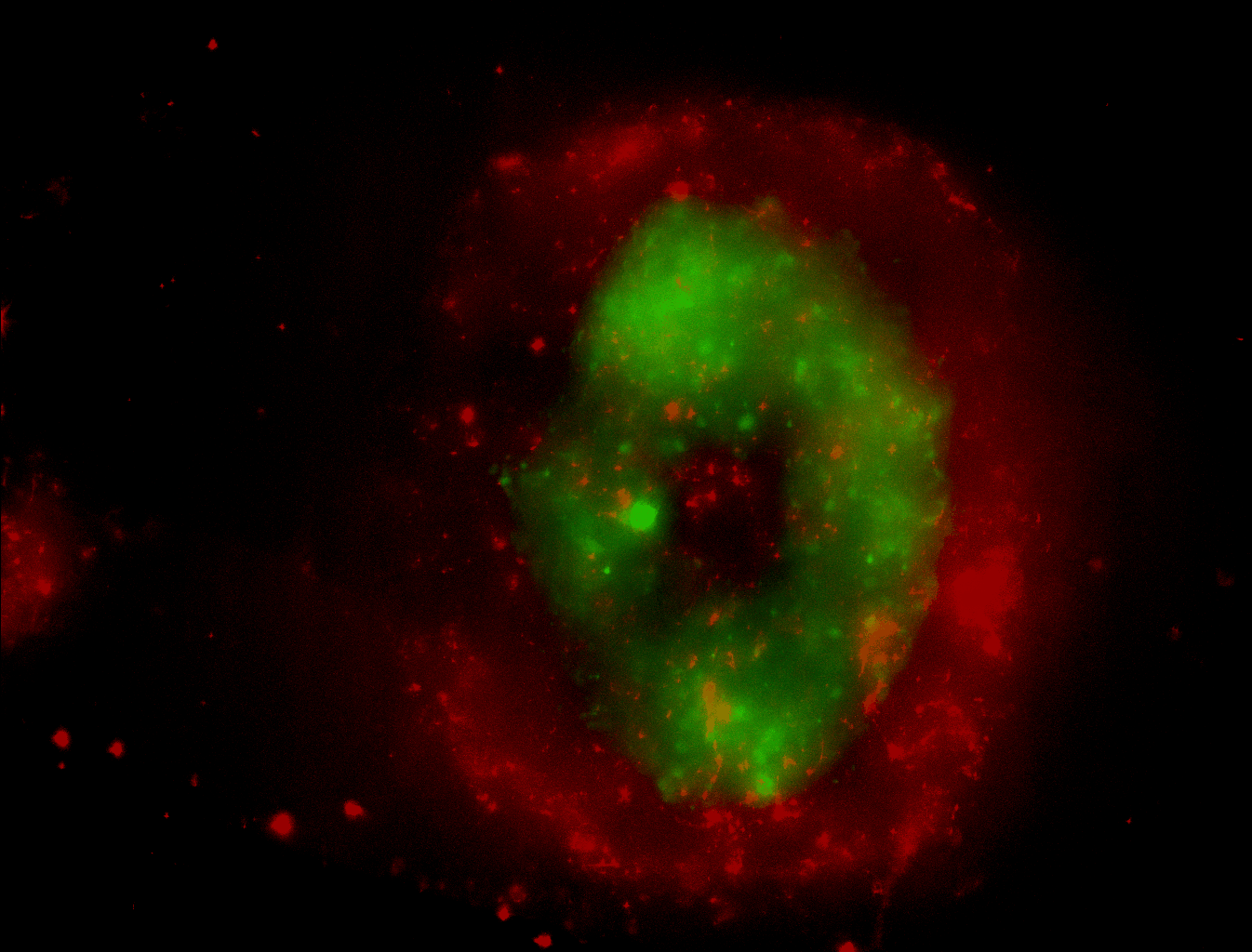

Supplement: Supplementary file 4 — Supplementary Material 4 [file 12967_2025_7307_MOESM4_ESM.gif]

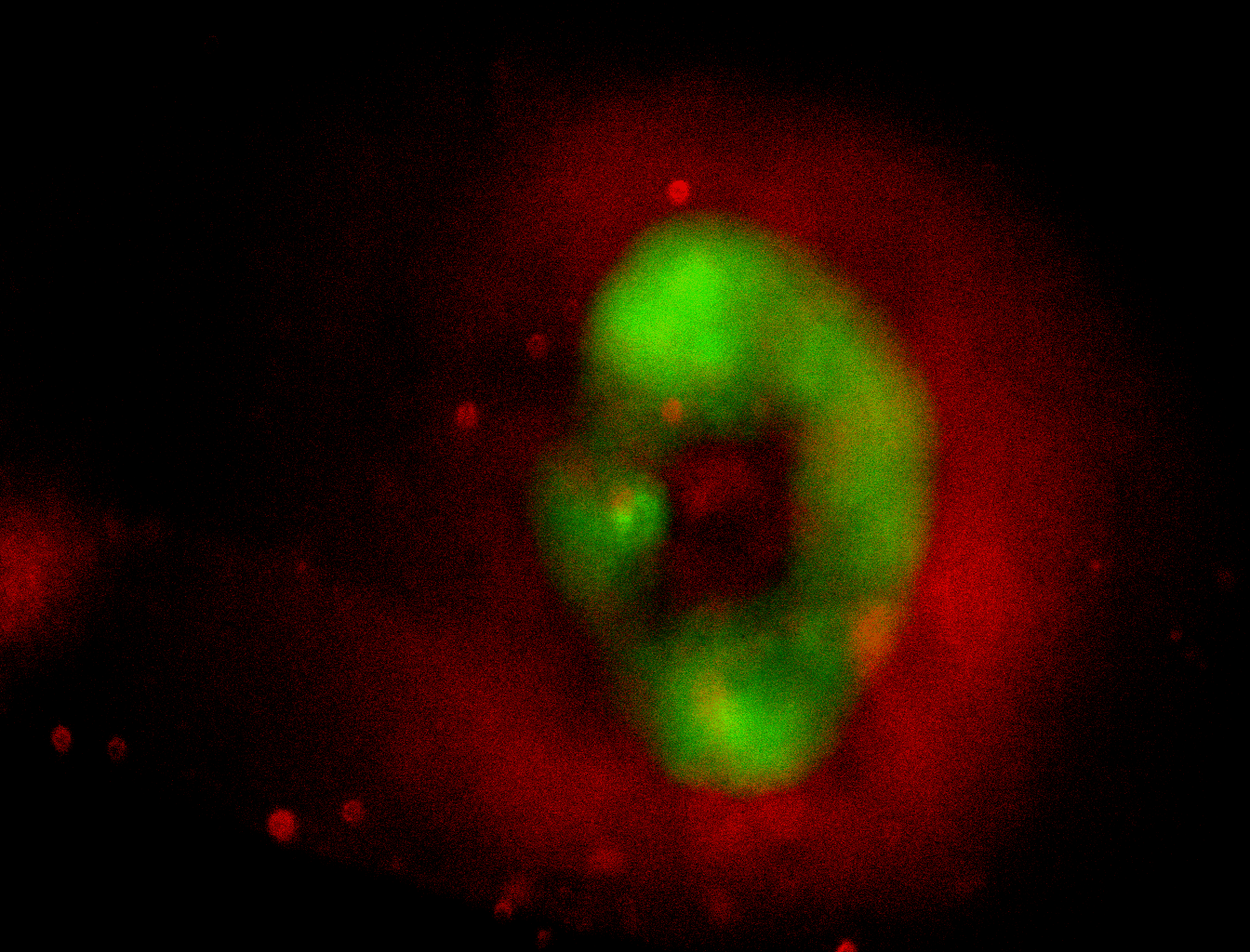

Supplement: Supplementary file 5 — Supplementary Material 5 [file 12967_2025_7307_MOESM5_ESM.gif]

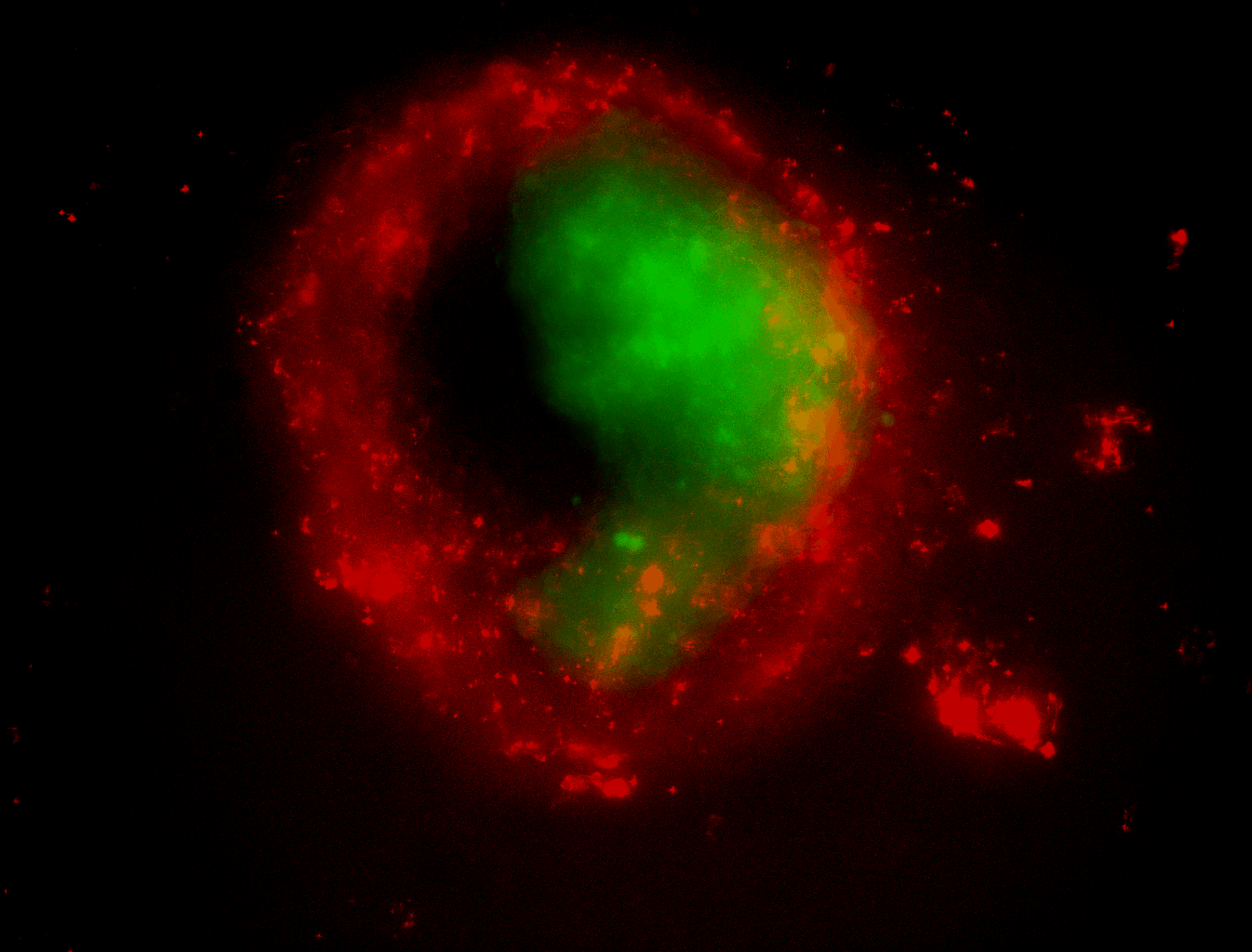

Supplement: Supplementary file 6 — Supplementary Material 6 [file 12967_2025_7307_MOESM6_ESM.gif]

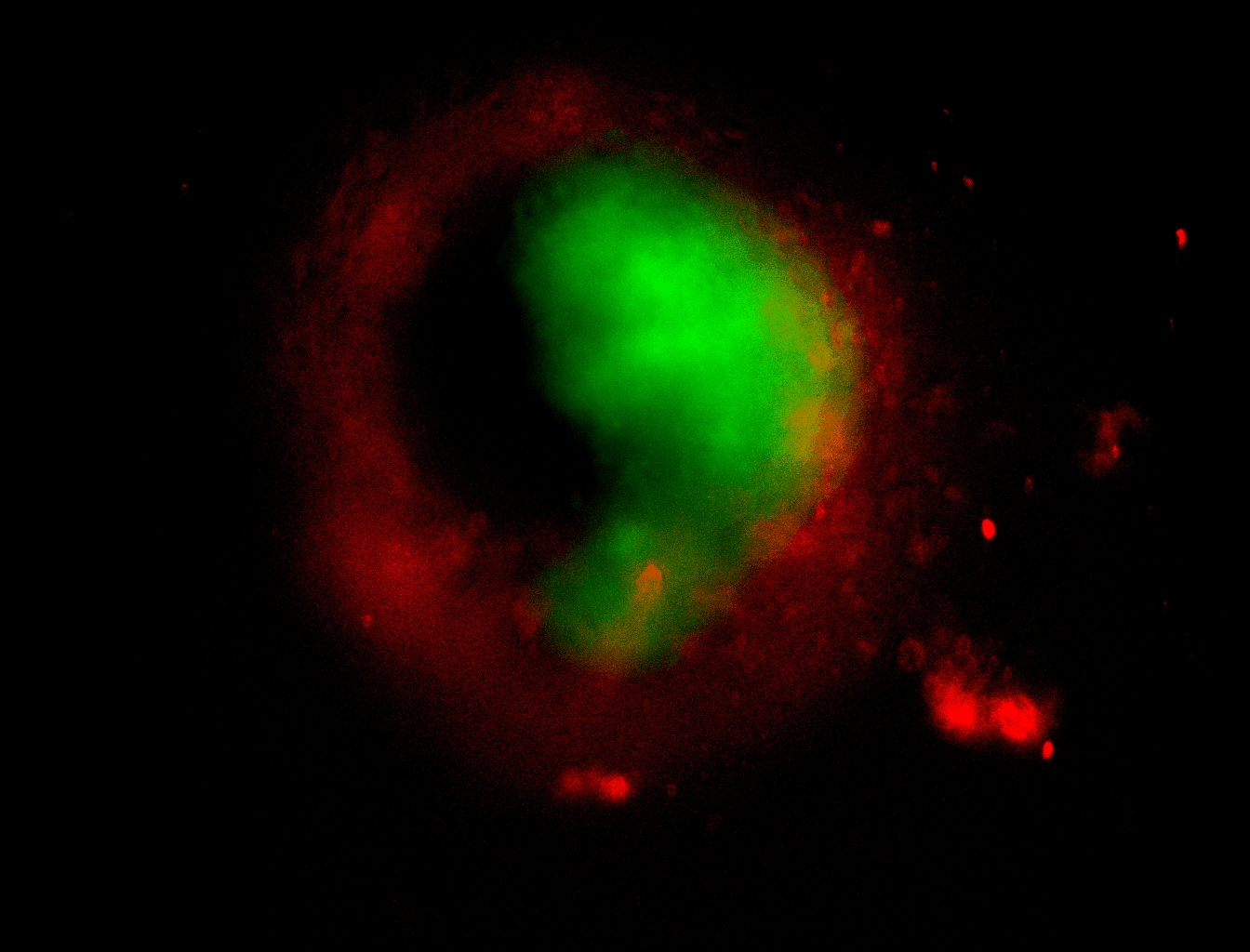

Supplement: Supplementary file 7 — Supplementary Material 7 [file 12967_2025_7307_MOESM7_ESM.gif]

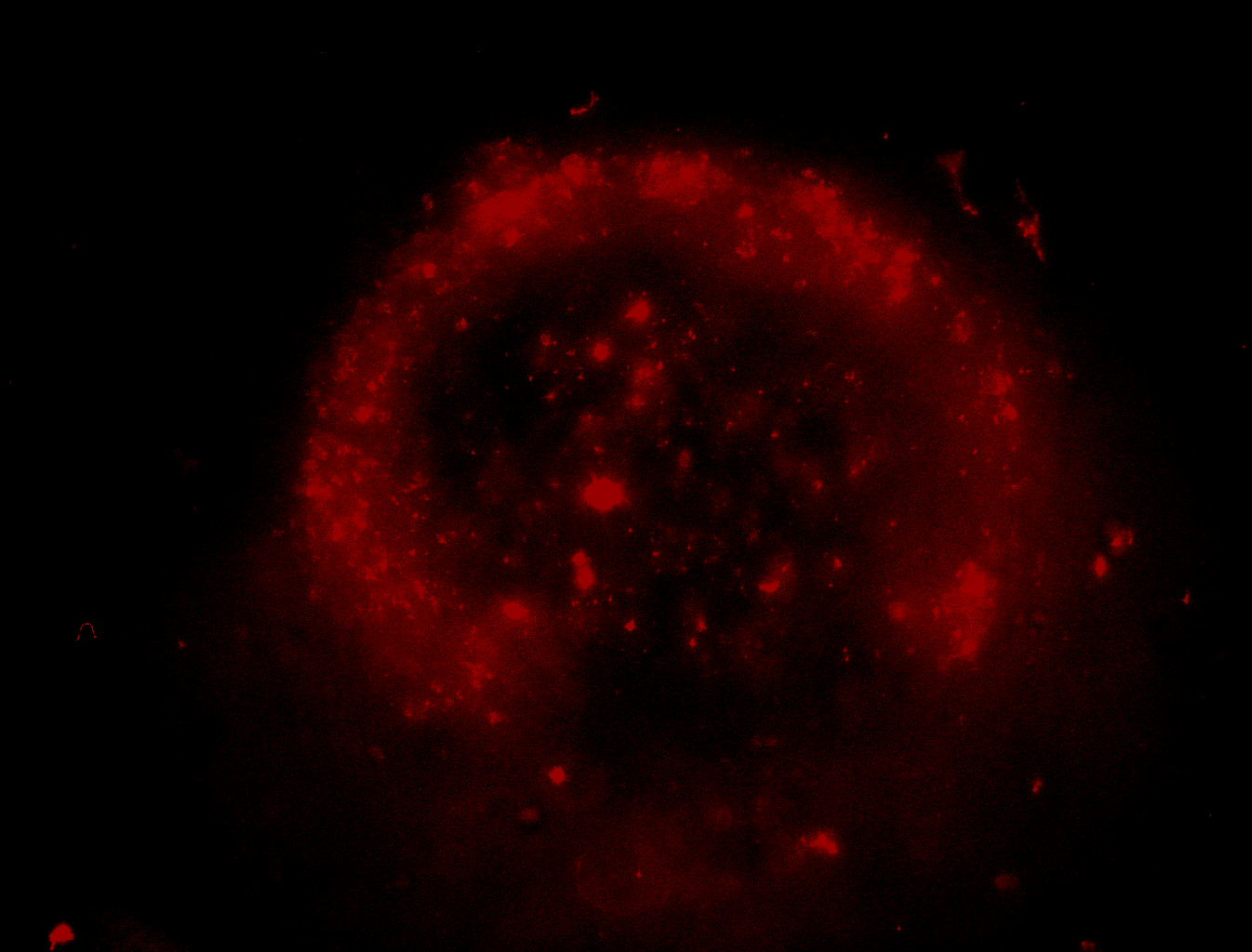

Supplement: Supplementary file 8 — Supplementary Material 8 [file 12967_2025_7307_MOESM8_ESM.gif]

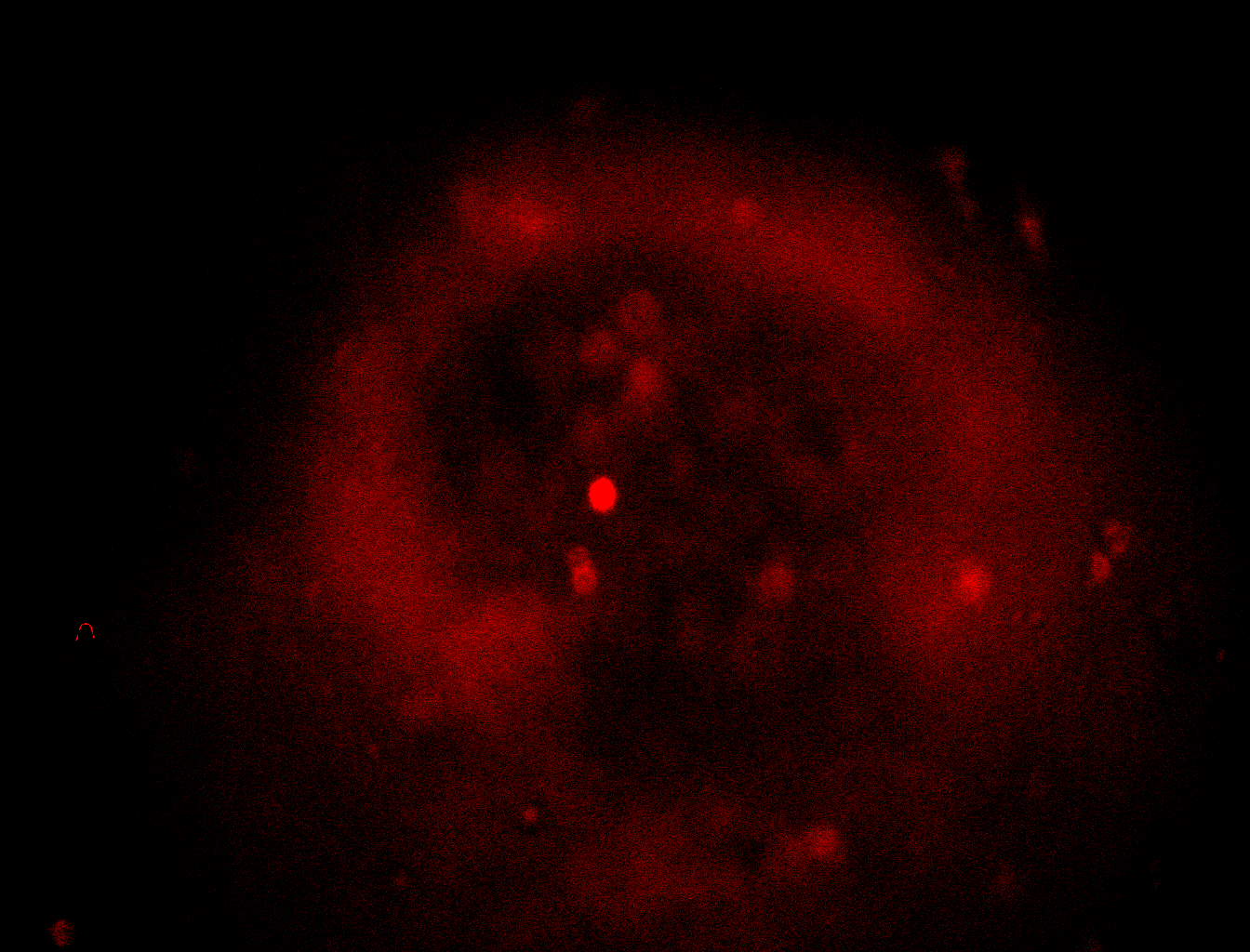

Supplement: Supplementary file 9 — Supplementary Material 9 [file 12967_2025_7307_MOESM9_ESM.gif]

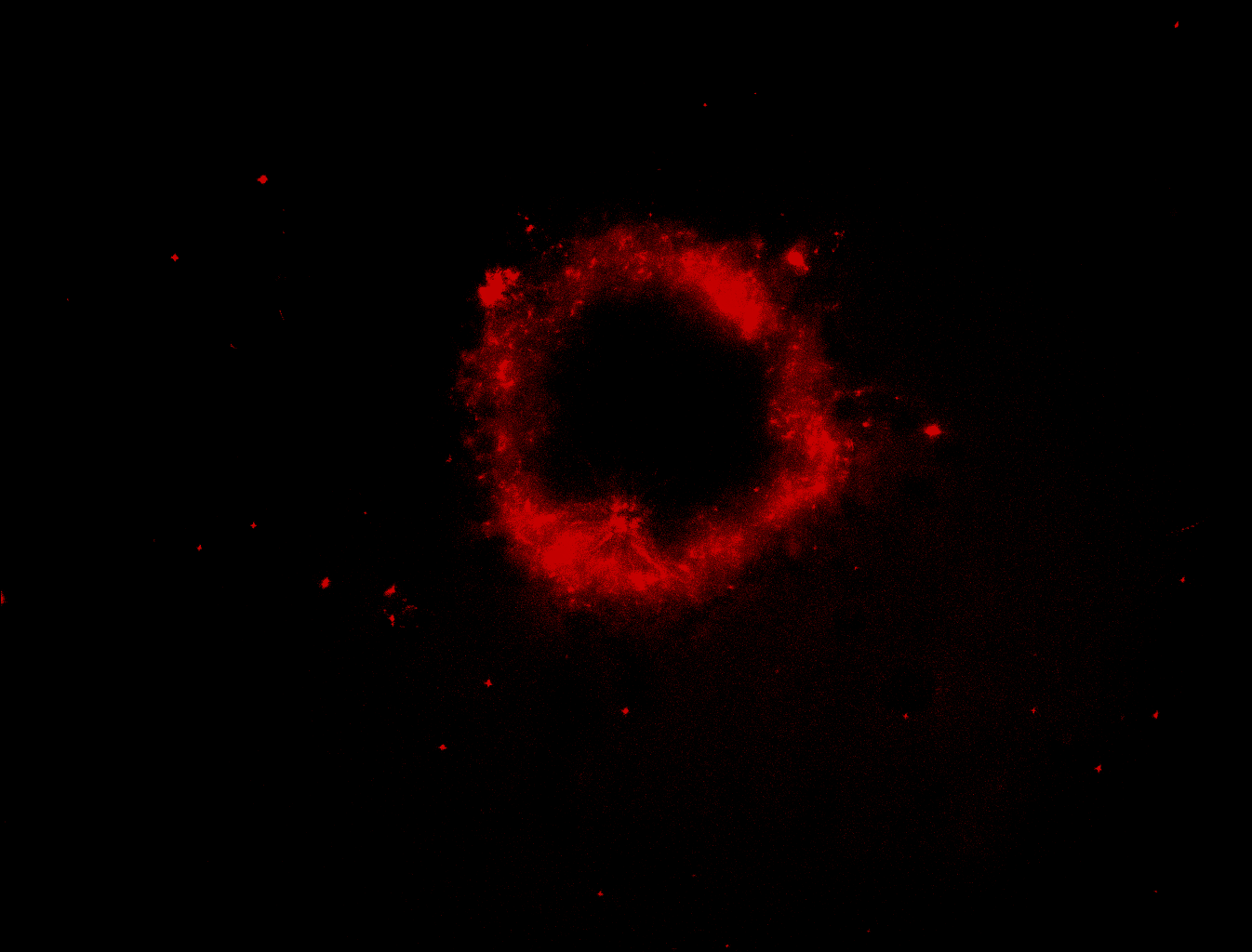

Supplement: Supplementary file 10 — Supplementary Material 10 [file 12967_2025_7307_MOESM10_ESM.gif]

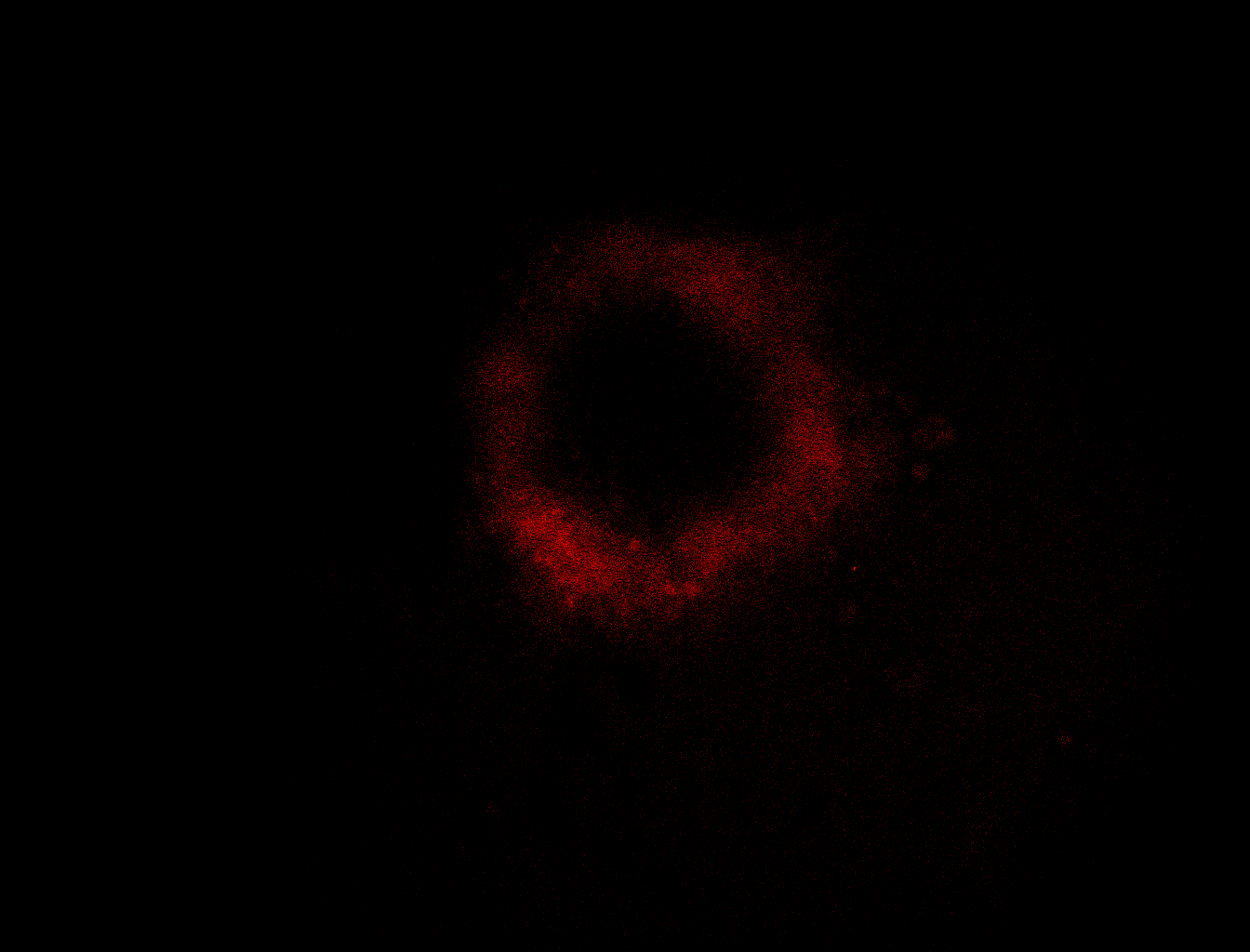

Supplement: Supplementary file 11 — Supplementary Material 11 [file 12967_2025_7307_MOESM11_ESM.gif]
