# Supplementary material for: Fibroblast activation protein (FAP)-mediated promotion of metastasis via the FN1-TGFβ axis and immune suppression in aggressive thyroid cancer
Source: J Transl Med. 2025 Nov 13;23:1284. doi: 10.1186/s12967-025-07307-3 (PMC12616960; doi:10.1186/s12967-025-07307-3)
Supplement: Supplementary file 13 — Supplementary Material 13 [file 12967_2025_7307_MOESM13_ESM.pdf]

Material	Company	Cat.No.
rabbit zenon 647 *	Invitrogen	Z25308
rabbit zenon 488 *	Invitrogen	Z25302
mouse zenon 488 *	Invitrogen	Z25002
mouse zenon 594 *	Invitrogen	Z25007
rabbit zenon 594 *	Invitrogen	Z25307
rabbit - FAP *	CellSignaling	66562S
rabbit - TGFβ *	CellSignaling	3711S
rabbit – pPLC-β3 *	CellSignaling	2484
rabbit - FN1 *	CellSignaling	26836S
Phospho-p44/42 MAPK (Erk1/2) (Thr202/Tyr204) *	CellSignaling	9101
Phospho-Akt (Ser473) (D9E) XP® rabbit mAb *	CellSignaling	4060
Phospho-Stat3 (Tyr705) (D3A7) XP® rabbit mAb *	CellSignaling	9145
GAPDH (14C10) rabbit mAb *	CellSignaling	2118
Rabbit anti-mouse IgG (HRP Conjugate) *	CellSignaling	58802
Mouse Anti-Rabbit IgG (HRP Conjugate) *	CellSignaling	93702
TEM8 405 **	Novus	5658AF 405
HLA ABC APC **	Biolegend	311410
HLA DP/DQ/DR APC cy7 **	Novus	64358 APC cy7
PDL1 PE **	Biolegend	124808
CD9 APC cy7 **	Abcam	AB239306
CD86 PE/Dazzle **	Biolegend	374218
aSMA APC **	RnD	IC1420A
CD29 PE **	RnD	FAB17781F
FITC anti-human HLA-A,B,C antibody **	Biolegend	311404
mouse - FAP **	RnD	MAB3715
APC Mouse IgG2a, κ isotype **	Biolegend	400219
Mouse IgG_1 Alexa Fluor® 405-conjugated antibody **	RnD	IC002V

APC-Cy™7 Mouse IgG2a, κ isotype **	BD bioscience	557751
PE Mouse IgG1, κ isotype **	Biolegend	400111
PE/Dazzle™ 594 Mouse IgG1, κ isotype **	Biolegend	981814
rabbit FAP 1:200 ***	abcam	AB207178
rabbit CD29 1:1000 ***	abcam	AB179471
Anti-MHC class I + HLA A + HLA B antibody 1:300 ***	abcam	ab134189
Anti-fibronectin antibody 1:250 ***	DAKO	A0245
Anti-PD-L1 antibody [28-8] 1:400 ***	CellSignaling	13684
CD8, clone C8/144B 1:200 ***	Agilent DAKO	M 7103
mouse aSMA 1:4000 ***	DAKO	M0851
rabbit c-myc1:50 ***	Cellmarque	395R-16
FITC anti-c-Myc antibody [Y69] **	abcam	ab223913
Invitrogen bolt 4%-12% Bis Tris Plus Western blot gels	Invitrogen	22122010
Taq Pro Universal SYBR qPCR Master Mix	VAZYME	Q712-03
SignalFire™ ECL reagent	CellSignaling	6883
VitroGel® Hydrogel Matrix	TheWell bioscience	VHM01
Antibodies for Western blot and/or live imaging *		
Antibodies for FACS **		
Antibodies for immunohistochemistry ***		
BioRAD CFXConnect qPCR	Biorad	
iBright 750	Invitrogen	
iBlot PVDF	Invitrogen	IB24002
iBlot Nitrocellulose	Invitrogen	IB25002
iBlot2 machine	Invitrogen	IB21001
TECAN Infinite M200 Pro	Tecan	30050303
EDTA Protease / Phosphatase 100x	Thermo scientific	78440
Bovine serum albumin Fraction V	Sigma-Aldrich	10735094001
LB Agar	Roth	x9692

Agarose	Serva	9012-36-6
RevertAid First strand cDNA synthesis kit	Thermo Fisher	k1622
fractionation kit	CellSignaling	9038S
recombinant FAP	Biolegend	768906
recombinant FN1	Biolegend	775308
Intracellular transcription factor buffer kit	BD Pharmigen	562574
Gibco RPMI 1640X	Gibco	29875-034
Gibco Opti med	Gibco	31985-047
Lonza EMEM	Lonza	12-125F
FETAL+	Anprotec	AC-5M-0161
L-Glutamine	Sigma-Aldrich	G7513
Pen/Strep antibiotics	Sigma-Aldrich	P0781
Trypsine	Gibco	15400-054
RNAiMAX	Invitrogen	13778-075
Lipofectamine 2000	Invitrogen	11668-027
siRNA FAP	Thermo scientific	s5023
siRNA negative control	Thermo scientific	AM4611
FAP-GFP overexpression plasmid	Origene	RG204692
pmR-mCherry vector negative control	632542	TAKARA
FAP inhibitor BR103354	Aobious	AOB14444
Ibidi culture µ-dish with cross	Ibidi	80466
Ibidi culture µ-slide +collagen I coat	Ibidi	80809
12-well transwell insert 0.4µm	Qart	335870
24-well transwell insert 8µm	CORNING	3464
RNA isolation kit	Machery-Nagel	740955 250
PIERCE BCA Protein kit	Thermo scientific	23225
Nucleo bond plasmid purification kit	Machery-Nagel	74041 250
Proteome Profiler Cytokine XL	RnD	ARY022B
Proteome Profiler Angiogenesis	RnD	ARY007

Proteome Profiler Phospho-Kinase	RnD	ARY003C
1-plex total/free active TGF-β1 detection kit	Biolegend	740450
β-mercaptoethanol	Biochemica	
recombinant Human TGF-β1	Biolegend	781802
Gel loading dye 6x	England biolabs	10127723
Page ruler Western blot ladder	Thermo scientific	1194444
OLIGO TARGET	**Sequence (5'->3')**	
PDL1 Forward primer	TGCCGACTACAAGCGAATTACTG	
PDL1 Reverse primer	CTGCTTGTCCAGATGACTTCGG	
HLA-A Forward primer	AGATACACCTGCCATGTGCAGC	
HLA-A Reverse primer	GATCACAGCTCCAAGGAGAACC	
HLA-B Forward primer	CTGCTGTGATGTGTAGGAGGAAG	
HLA-B Reverse primer	GCTGTGAGAGACACATCAGAGC	
HLA-C Forward primer	GGAGACACAGAAGTACAAGCGC	
HLA-C Reverse primer	ACATCCTCTGGAGGGTGTGAGA	
FGF2 Forward primer	AGCGGCTGTACTGCAAAAACGG	
FGF2 Reverse primer	CCTTTGATAGACACAACTCCTCTC	
VIM Forward primer	AGGCAAAGCAGGAGTCCACTGA	
VIM Reverse primer	ATCTGGCGTTCCAGGGACTCAT	
FN1 Reverse primer	GGACACAACGATGCTTCCTGAG	
SNAI2 Forward primer	CCCTCACTGCAACAGAGCAT	
ZEB1 Forward primer	GGCATACACCTACTCAACTACGG	
ZEB1 Reverse primer	TGGGCGGTGTAGAATCAGAGTC	
ACTA2/aSMA Reverse primer	CAGATCCAGACGCATGATGGCA	
CDH2 Forward primer	CCTCCAGAGTTTACTGCCATGAC	
FAP Forward primer	GGAAGTGCCTGTTCCAGCAATG	
FAP Reverse primer	TGTCTGCCAGTCTTCCCTGAAG	

S1. Materials and equipment used for this research publication and primers sequences
